# The Unconventional Role of ABHD17A in Increasing the S-Palmitoylation and Antiviral Activity of IFITM1 by Downregulating ABHD16A

**DOI:** 10.3390/biom15070992

**Published:** 2025-07-11

**Authors:** Xuemeng Shi, Shuaiwu Chen, Mingyang Liu, Yali Fan, Xin Wen, Jingyi Wang, Xiaoling Li, Huimin Liu, Lin Mao, Li Yu, Yuxin Hu, Jun Xu

**Affiliations:** College of Life Science, Henan Agricultural University, Zhengzhou 450046, China; xmshi@henau.edu.cn (X.S.); chenshuai_wu@163.com (S.C.); 15518250709@163.com (M.L.); 15539816516@163.com (Y.F.); wenxin@henau.edu.cn (X.W.); wangjingyi2040@126.com (J.W.); lixiaolingcell@163.com (X.L.); liuhuimin@henau.edu.cn (H.L.); limmao@henau.edu.cn (L.M.); yl2251680499@163.com (L.Y.); 15936658923@163.com (Y.H.)

**Keywords:** ABHD17A, IFITM, S-palmitoylation, post-translational modification, virus infection

## Abstract

The broad-spectrum antiviral functions of interferon-inducible transmembrane 1 (IFITM1) rely on S-palmitoylation post-translational modification. α/β-hydrolase domain-containing 17A (ABHD17A) has been reported to be responsible for protein depalmitoylation over the past decade, but whether and how ABHD17A regulates the dynamic S-palmitoylation modification of IFITM1 remains unknown. Here, we demonstrated that ABHD17A physically interacts with IFITM1 and increases the S-palmitoylation level of IFITM1. Sequence alignment revealed that ABHD17A lacked the DHHC motif, which is capable of catalyzing the S-palmitoylation modification. Thus, we screened multiple candidate palmitoylating and depalmitoylating enzymes that may contribute to ABHD17A-induced upregulation of IFITM1 S-palmitoylation. The recently discovered depalmitoylase ABHD16A was significantly downregulated by ABHD17A, which counteracted the palmitate-removing reactions of ABHD16A on IFITM1 and subsequently upregulated the S-palmitoylation level and antiviral activity of IFITM1. Our work therefore elucidated the unconventional role of depalmitoylase ABHD17A in elevating the S-palmitoylation modification, expanded the biological functions of ABHD17A in innate immunity, and provided potential targets for viral disease therapy.

## 1. Introduction

The interferon-induced transmembrane (IFITM) members are small homologous proteins that can be rapidly induced by interferons (IFNs) and are evolutionarily conserved among vertebrates [1]. Five IFITM homologs have been identified in humans, among which IFITM1 is primarily distributed on the plasma membrane and restricts viral invasion by restricting the hemifusion between viral and host cell membranes [2,3]. In the past decades, we and other groups have reported that the plasma membrane distribution and broad-spectrum antiviral activity of IFITM1 rely on S-palmitoylation post-translational modification [4,5,6,7,8]. However, studies on the palmitoyl acyltransferase and depalmitoylase responsible for the dynamic S-palmitoylation modification of IFITM1 are very limited.

S-palmitoylation belongs to S-acylation [9], typically referring to the covalent binding of saturated palmitic acids to specific cysteines through thioester bonds, which can exert multiple effects in regulating virus–host interaction [10]. The palmitoyl acyltransferases (PATs) responsible for catalyzing the connection of palmitic acid groups to cysteines in humans include twenty-three zinc finger DHHC domain-containing (zDHHC) members [11]. McMichael et al. once screened and attempted to find PATs that can target IFITM proteins for palmitoylation modification. Thirteen zDHHCs have been identified to elevate the S-acylation level of IFITM3 to varying degrees, but only overexpression of zDHHC20 had the most robust activity and facilitated IFITM3′s resistance to influenza A virus infection [12]. In addition, we have preliminarily discovered that zDHHC3 increased the S-palmitoylation modification level of IFITM1 in porcine kidney epithelial cells, which may contribute to swine IFITM1 resisting Japanese encephalitis virus (JEV) replication [6]. However, it remains unclear how the S-palmitoylation modification of human IFITM1 is achieved and which PAT will be involved in the catalytic process.

S-palmitoylation is reversible, with depalmitoylase catalyzing the hydrolysis of thioesters to remove the palmitate from substrate proteins [13]. Acyl-protein thioesterases (LYPLA1 and LYPLA2) [14,15], palmitoyl protein thioesterases (PPT1 and PPT2) [16], and three α/β-hydrolase domain (ABHD)-containing family members (ABHD17A, ABHD10, and ABHD16A) have been categorized as depalmitoylases and play pivotal roles in cellular signaling, metabolism, and innate immunity [4,5,17,18]. To identify the depalmitoylase targeting for IFITM1, Lehner et al. have conducted a high-throughput yeast two-hybrid screen system, which suggested ABHD16A may interact with IFITM1 [19]. Subsequently, we predicted that ABHD16A may be a potential depalmitoylase by analyzing its sequence and structure [20]. In 2022, we demonstrated that ABHD16A regulates the antiviral invasion of IFITM1 through specific depalmitoylation of IFITM1 in humans, swine, and mice [5], which fills the gap in the depalmitoylation reaction of IFITM1.

In the past decade, ABHD17A has been reported to deacylate N-Ras [17,21], postsynaptic density (PSD)-95 [22], and the stress axis-regulated exon (STREX) of ion channels [23]. Importantly, emerging evidence underscores the significance of ABHD17A in innate immunity. Zheng et al. discovered that the depalmitoylation of pyrin domain (PYD)-containing protein 3 (NLRP3) inflammasome was catalyzed by ABHD17A [24]. In addition, Dixon et al. recently demonstrated that inhibiting ABHD17A led to the increased plasma membrane localization and S-palmitoylation of nucleotide-binding oligomerization domain-containing protein 2 (NOD2) [25], which acts as an innate immune receptor to sense bacterial peptidoglycan-conserved motifs [26]. Nevertheless, at present, studies on the role of ABHD17A in viral infection processes remain unclear. As a classic depalmitoylase, we are curious whether ABHD17A can target IFITM1 to participate in regulating virus–host interactions.

In this study, we demonstrated that ABHD17A did not remove the palmitate of IFITM1, but significantly upregulated the S-palmitoylation modification level of IFITM1. Importantly, ABHD17A altered the expression of multiple palmitoylases and depalmitoylases, among which the depalmitoylase ABHD16A targeting IFITM1 was specifically downregulated by ABHD17A. This effect led to the weakening of depalmitoylation of IFITM1, which in turn explained why ABHD17A upregulated the S-palmitoylation and antiviral capacity of IFITM1. Together, our findings highlight the unconventional machinery by which ABHD17A facilitates the S-palmitoylation of IFITM1 to inhibit various viral infections, which also reveals a previously unknown virological function of ABHD17A in innate immunity.

## 2. Materials and Methods

### 2.1. Cell Culture and Transient Transfection

The HEK293 (#CRL-1573) human embryonic kidney cells, A549 (#CCL-185) human non-small cell lung cancer cells, and NIH/3T3 (#CRL-1658) mouse embryonic fibroblast cells were derived from the American Type Culture Collection (ATCC). The HepG2.215 (RN-53413) human hepatoma cells were purchased from Noangene (Wuhan, China). All cells were cultured in high glucose Dulbecco’s modified Eagle’s medium (DMEM; #11995065; Gibco, Waltham, MA, USA), supplemented with 10% heat-inactivated fetal bovine serum (FBS; #F0850; Merck, St. Louis, MO, USA), 10 U/mL penicillin, 10 μg/mL streptomycin, and 4 mM L-Glutamine at 37 °C in humidified atmosphere with 5% CO_2_. Cells were transiently transfected with Lipofectamine 3000 (#L3000001; Invitrogen, Carlsbad, CA, USA) at a 3:1 Lipofectamine-to-DNA ratio.

### 2.2. Plasmid Construction

The coding sequences of human ABHD17A, IFITM1, zDHHC1, zDHHC20, zDHHC24, and ABHD16A were amplified from total RNA isolated from HEK293 cells. The target cDNA fragments were digested with restriction endonucleases and, respectively, inserted into pDsRed-monomer-N1, pEGFP-N1, pcDNA3.1(+)-3×Flag, and pcDNA3.1(+)-HA vectors. All plasmids were sequenced for verification.

### 2.3. Immunofluorescence (IF)

HEK293 cells were fixed with 3.7% paraformaldehyde (PFA) for 20 min, permeabilized with 0.2% Triton-X100 for 5 min, blocked with 5% BSA for 20 min, and then incubated with ABHD17A/IFITM1/JEV-E antibody for 2 h and SAlexa Fluor 488/594-conjugated secondary antibody for 1 h (#K0031G-AF488/#K1034G-AF594; Solarbio, Beijing, China). Afterwards, coverslips were mounted with antifade mounting medium. Leica TCS SP8 laser scanning confocal microscope equipped with a 60×/1.5 oil lens objective (Nikon, Tokyo, Japan) was used to acquire images.

### 2.4. Bimolecular Fluorescence Complementation Assay (BiFC)

BiFC, a technique that utilizes the characteristics of fluorescent proteins such as Venus (a variant of yellow fluorescent protein, YFP) to study protein interactions [27]. Venus can be separated from the amino acid position 173, producing two non-fluorescent active fragments (VN: 1–173 aa; VC: 174–239 aa). When VN and VC were fused to interacting proteins, they would be pulled closer and complemented due to the interaction of the proteins to rebuild into active fluorescent proteins and produce fluorescence under excitation light. Briefly, the cDNA sequences of ABHD17A and IFITM1 were, respectively, inserted into the pVN vector expressing the 1–173 amino acids of Venus and the pVC vector expressing the 174–239 amino acids of Venus. HEK293 cells were introduced with the following plasmids: pVN + pVC, or ABHD17A-VN + IFITM1-YC. After 24 h post-transfection, cells were viewed using a Leica SP8 confocal microscopy (Nikon, Tokyo, Japan).

### 2.5. Yeast Two-Hybrid Assay (Y2H)

cDNA sequences of human ABHD17A and IFITM1 were cloned into the bait vector pGBKT7 with the Gal4-binding domain (BD) and the prey vector pGADT7 expressing the activation domain (AD), respectively. The AH109 yeast colonies were cultured in YPDA medium (2% Tryptone, 1% Yeast extract, 2% Glucose, 0.02% Adenine). Transform the recombinant plasmid into yeast cells, transfer yeast cells to synthetic dropout (SD) medium: SD-Trp/-Leu (SD-TL) plates for amplification, and then spread them onto SD-Trp/-Leu/-His (SD-TLH) medium for screening. Finally, the yeast were transferred to SD-Trp/-Leu/-His/-Ade (SD-TLHA) containing X-α-Gal and left at 30 °C for 3–4 days to confirm protein interactions by examining the blue colonies.

### 2.6. Western Blotting (WB)

RIPA lysis buffer supplemented with protease inhibitor cocktail (#P8340; Merck, St. Louis, MO, USA) was used to harvest cell lysates. Equal amounts of whole cell lysates were incubated with SDS loading buffer, boiled at 98 °C for 10 min, and subjected to SDS-PAGE. Proteins were transferred to a 0.45 µm PVDF membrane, and the PVDF membrane was then blocked, and incubated with primary antibody for 12 h at 4 °C and corresponding secondary antibody for 2 h at room temperature, followed by visualization using ECL chemiluminescence. Antibodies used in this study were listed in Table S1.

### 2.7. Co-Immunoprecipitation (Co-IP)

Transfected HEK293 or A549 cells were lysed in Triton X-100 buffer containing 20 mM Tris, pH 7.4, 1% Triton-X100, 150 mM NaCl, with 1% PMSF. Then, 10% of the cell lysates were kept as the input, and the rest of the lysates were incubated with the Protein A + G magnetic beads (#P2108; Beyotime, Shanghai, China) bound with anti-Flag at 4 °C overnight. Consequently, magnetic separation rack was utilized to collect the magnetic beads, and incubated with 3×Flag Peptide for 2 h to competitively wash out the Flag-tagged proteins with their complexes and eliminate the influence of IgG heavy and light chains. The supernatant was subjected to SDS-PAGE. To verify the interplay between endogenous ABHD17A and IFITM1, normal mouse IgG and anti-IFITM1 were used to immunoprecipitate endogenous ABHD17A in HEK293 cells.

### 2.8. GST-ABHD17A Expression and Purification

Briefly, we cloned the cDNA fragment of human ABHD17A into the pGEX-4T-1 vector, and transformed it into *E. coli* BL21(DE3). Next, 1 mM IPTG was used to induce the expression of GST-tagged ABHD17A for 20 h at 16 °C. The protein was captured by GST-tag purification resin and eluted with GSH buffer (50 mM Tris, 150 mM NaCl, 10 mM GSH, pH 8.0). The concentration of purified protein was measured with a BCA Protein Assay Kit, and then adjusted to 1 mg/mL. In practical operation, about 1.8 mg of recombinant ABHD17A can be purified from every 500 mL of BL21 bacterial fluid.

### 2.9. GST Pull-Down

In brief, 20 μg of GST or GST-tagged ABHD17A was incubated with Glutathione Sepharose beads at 4 °C for 4 h. A total of 100 μg human His-IFITM1 recombinant bait proteins (#Ag16672; Proteintech, Rosemont, IL, USA) were added and rotated for 2 h at 4 °C. The beads were subsequently washed three times, and bound proteins were mixed with SDS loading buffer, boiled at 98 °C for 5 min, and subjected to Coomassie Blue staining to determine the interaction.

### 2.10. Quantitative RT-PCR (qRT-PCR)

Cells were lysed in RNA-easy Isolation Reagent (#R701-01; Vazyme, Nanjing, China). cDNA syntheses were conducted using HiScript II Q RT SuperMix Kit (#R223-01; Vazyme). qRT-PCR was performed using the StepOne Plus Real-Time PCR System (Applied Biosystems, Waltham, MA, USA) with HQ SYBR qPCR Mix (Without ROX) (#ZF501-1; Zoman Biotechnology, Beijing, China). Relative mRNA level was calculated by using 2^−ΔΔCt^ method by normalizing to the expression of GAPDH. Each experiment was tested in triplicate. Primers used are indicated in Table S2.

### 2.11. Luciferase Reporter Assay

To construct the human ABHD16A promoter luciferase vector, we cloned the ABHD16A promoter (1026 bp before the start codon) into the pGL3-basic through KpnI and HindIII sites. The pGL3-ABHD16A-promoter plasmid was introduced into HEK293 cells. For adherent HEK 293 cells grown in a 96-well plate, aspirate the cell culture medium and add 100 μL lysis buffer (#11401ES76; Yeasen, Shanghai, China). Gently rotate the 96-well plate until the lysis buffer completely covers the cells. Incubate the sample on ice for 5 min to fully lyse the cells. To measure the luciferase activity, 20 μL of cell lysate was mixed with 100 μL of luciferase assay reagent and then measured using the SpectraMax L Microplate Luminometer (San Jose, CA, USA). Readings in the luminometer were taken over a spectral wavelength range of 350–700 nm and an integration time of 5 s at room temperature. Each independent experiment was performed in quintuplicate.

### 2.12. Short Hairpin RNA (shRNA) Interference Experiment

The 21-nucleotide targeting human ABHD17A gene (5′-agatgagcagcttctacattg-3′) was designed and cloned into pTSB-SH-Fluor-2A-ARGs vector via AgeI and EcoRI sites. HEK293 cells were transfected with scrambled negative control (5′-ttctccgaacgtgtcacgt-3′) and ABHD17A shRNA plasmid for at least 48 h. Knockdown of ABHD17A expression efficiency was determined by Western blotting.

### 2.13. CRISPR/Cas9-Mediated Knockout of ABHD16A

These assays were performed as previously described [5]. Guide RNA targeting exon 3 of human ABHD16A was synthesized (F: 5′-caccgtcgccttcttctacttgtac-3′; R: 5′-aaacgtacaagtagaagaaggcgac-3′). The sgRNA sequences were inserted into LentiCRISPRv2 via BsmBI sites. The recombinant plasmid was introduced into HEK293 cells. An amount of 2 μg/mL puromycin dihydrochloride was added to screen ABHD16A knockout cells for 28 days. Limited dilution culture method was used to screen monoclonal cells. Once the monoclonal cell line is confirmed, we will no longer add puromycin to the culture medium.

### 2.14. Acyl-PEGyl Exchange Gel Shift Assay (APEGS)

The APEGS experiment was described by Percher et al. [28], and optimized by us [4,5,29]. Briefly, transfected HEK293 or A549 cells were harvested and lysed with lysis buffer (pH 7.3, 50 mM triethanolamine, 150 mM NaCl, 5 mM EDTA, 4% SDS, and 1 µg/mL leupeptin, pepstatin, and aprotinin) at 4 °C for 0.5 h. Then, 200 mM tris-(2-carboxyethyl) phosphine (TCEP) was added to reduce disulfide bonds at 55 °C for 1 h. Subsequently, 1 M N-ethyl maleimide (NEM) was added to block non-palmitoylated cysteines at 25 °C for 4 h. Samples were recovered twice with chloroform–methanol precipitation (methanol–chloroform: H_2_O = 4:1.5:3) and dissolved with TEA buffer (4% SDS, 4 mM EDTA). A total of 1 M NH_2_OH was further added for 4 h at 25 °C. The cysteines were replaced by 5 kDa PEG-mal at 25 °C for 4 h.

### 2.15. Virus Preparation and Infection

The stock of JEV SA14-14-2 live vaccine strain and VSVΔG pseudotypes were described previously [5]. In the viral infection assay, HEK293 or A549 cells were infected with viruses in a DMEM medium without serum for 2 h. Afterwards, cells were washed with DMEM twice, and the medium containing 2% FBS was added.

### 2.16. Enzyme-Linked Immunosorbent Assay (ELISA) of Secreted Hepatitis B e Antigen (HbeAg) and Hepatitis Surface Antigen (HbsAg)

Kits of HBeAg (#MM-62949H2, MeiMian, Yancheng, China) and HbsAg (#MM-62949H1, MeiMian, Yancheng, China) were used to check the secreted HbeAg and HbsAg in HepG2.215 cell culture medium, respectively. OD 450 nm was set to obtain the absorption value.

### 2.17. Extracellular HBV DNA Extraction

Genomic DNA Purification Kit (#2868751, Thermo, Waltham, MA, USA) was used to extract the extracellular HBV DNA in HepG2.215 cell culture medium.

### 2.18. Cell Viability Analysis

Cell proliferation was measured by using the Enhanced Cell Counting Kit-8 (#C0041; Beyotime, Shanghai, China). Usually, 100 µL of 2000 cells are added to each well of a 96-well plate. After cell adhesion, 10 µL of CCK-8 solution was added to each well and incubated at 37 °C for 1 h. OD 450 nm was set to obtain the absorption value.

### 2.19. Image Processing and Analysis

ImageJ 1.54 p (National Institutes of Health, Bethesda, MD, USA) was used to adjust the brightness and contrast of images. A 1.5-pixel-wide median filter, a 2-pixel-wide unsharp mask, and a 30-pixel-wide rolling-ball background subtraction were utilized to elevate the signal-to-noise ratio. To determine the colocalization within cells, we used the ImageJ RG2B colocalization plugin to label the colocalization signals.

### 2.20. Quantification and Statistical Analysis

Results were represented as mean ± SD. Unpaired two-tailed *t*-test and one-way ANOVA followed by Tukey’s post hoc test was used to evaluate the significant differences between two groups and three or more groups using GraphPad Prism 9.5 (GraphPad Software, San Diego, CA, USA), respectively. ns, not significant, * *p* < 0.05, ** *p* < 0.01, *** *p* < 0.001. Experiments were repeated independently at least three times.

## 3. Results

### 3.1. Identification of IFITM1 as ABHD17A-Interacting Protein

**Figure 1:**
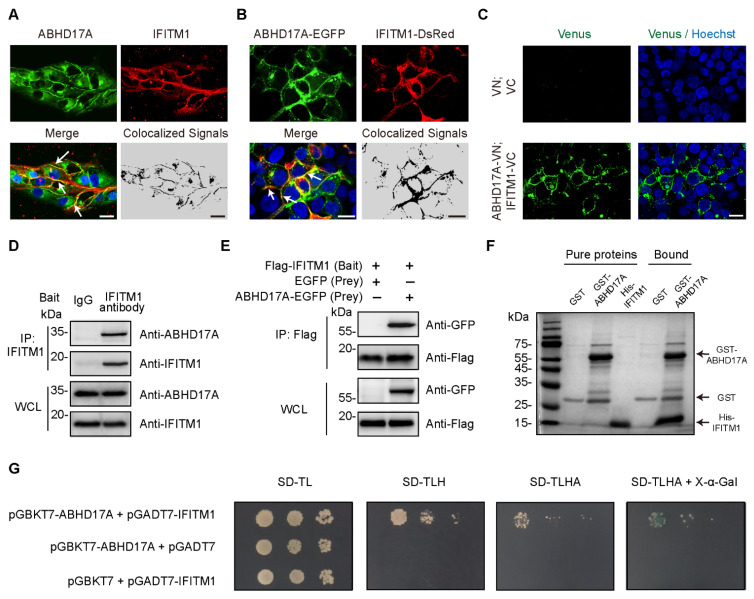
Identification of the interaction between ABHD17A and IFITM1. (**A**) Localization of endogenous ABHD17A and IFITM1 in HEK293 cells was detected with the corresponding primary antibodies. White arrows denoted ABHD17A and IFITM1 colocalized on the plasma membrane. Colocalized signals were analyzed via the ImageJ RG2B plugin. Scale bar, 20 μm. (**B**) Representative confocal micrographs showing the plasma membrane localization of exogenous ABHD17A with IFITM1. HEK293 cells were transfected with expression plasmids of ABHD17A-EGFP and IFITM1-DsRed. Then, 24 h after transfection, cells were stained with Hoechst 33342, and subjected to confocal microscopy. Scale bar, 20 μm. (**C**) Interaction of ABHD17A and IFITM1 in living cells. HEK293 cells were transiently transfected with the indicated plasmids. After 24 h, cell nuclei were stained with Hoechst 33342 and subjected to confocal microscopy. VN (1 to 173 aa of Venus) and VC (174 to 239 aa of Venus). Scale bar, 20 µm. (**D**) The interaction between endogenous ABHD17A and IFITM1 was confirmed by Co-IP assay in HEK293 cells; IgG and anti-IFITM1 were, respectively, used as negative control and bait antibody for immunoprecipitation (IP). (**E**) HEK293 cells were transfected with expression plasmids encoding EGFP-N1 or ABHD17A-EGFP along with plasmid encoding Flag-IFITM1. Next, 24 h post-transfection, immunoprecipitation was performed with the Protein A + G magnetic beads binding with Flag antibody, and the whole-cell lysates (WCLs) and IPs were analyzed by Western blotting. (**F**) GST pull-down showing the physical interaction between ABHD17A with IFITM1. Glutathione beads loaded with GST or GST-ABHD17A were incubated with purified His-IFITM1. Eluted proteins were separated on the SDS-PAGE gel and analyzed by Coomassie blue staining. (**G**) Yeast two-hybrid (Y2H) to detect the interaction of ABHD17A with IFITM1. pGBKT7-ABHD17A/pGADT7-IFITM1, pGBKT7-ABHD17A/pGADT7, or pGBKT7/pGADT7-IFITM1 were transformed into yeast cells. Yeast cells were then cultivated in selection-deficient culture media.

It is well known that IFITM1 is localized mostly on the plasma membrane [2], but the subcellular distribution pattern of ABHD17A remains poorly understood. To assess this, we, respectively, started immuno-fluorescence and live-cell imaging experiments by examining the endogenous and exogenous localization relationship in HEK293 cells. Surprisingly, substantial colocalization between ABHD17A with IFITM1 was observed on the plasma membrane (Figure 1A,B). Furthermore, we investigated whether ABHD17A interacts with IFITM1 by using the bimolecular fluorescence complementation (BiFC) system in living cells. Most interaction signals (indicated by Venus) were detected on the plasma membrane (Figure 1C), which is consistent with the plasma membrane-associated colocalization of ABHD17A with IFITM1.

To further confirm the interaction between ABHD17A with IFITM1, we conducted Co-IP experiments on both endogenous and exogenous expression of ABHD17A and IFITM1 in HEK293 and A549 cells. By using either IFITM1 or the Flag bait antibody, ABHD17A was readily co-immunoprecipitated (Figure 1D,E and Figure S1A). Furthermore, to determine whether ABHD17A directly interacts with IFITM1, the in vitro GST pull-down and yeast two-hybrid (Y2H) assay were conducted, which indicated ABHD17A physically interacts with IFITM1 (Figure 1F,G). Together, these results demonstrated that ABHD17A colocalized and directly interacted with IFITM1, which indicated the potential regulation of ABHD17A to IFITM1.

### 3.2. The Unconventional Role of ABHD17A in Increasing the S-Palmitoylation Modification of IFITM1

**Figure 2:**
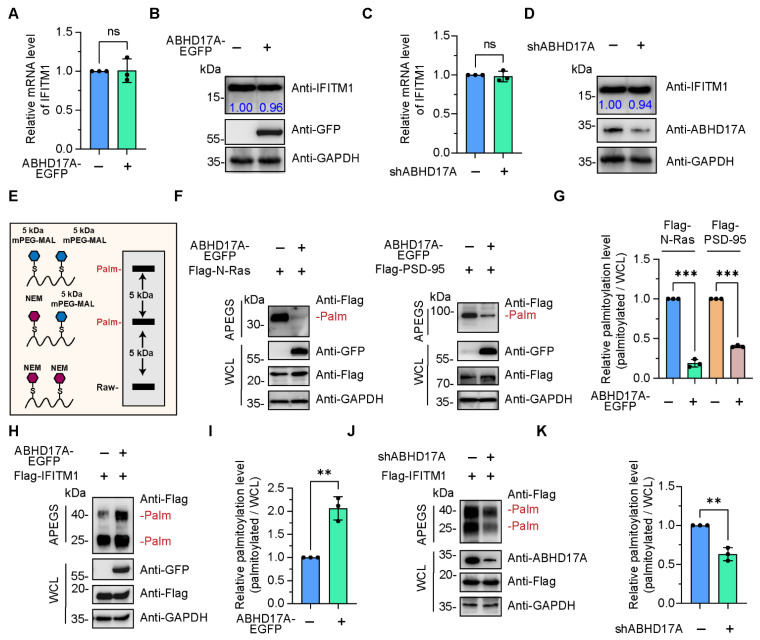
ABHD17A enhances the S-palmitoylation level of IFITM1. (**A**) The mRNA levels of IFITM1 in EGFP or ABHD17A-EGFP-expressing HEK293 cells were analyzed by qRT-PCR. (**B**) HEK293 cells were introduced with EGFP or ABHD17A-EGFP. At 24 h post-transfection, cell lysates were analyzed by Western blotting assays. The gray value of endogenous IFITM1 was quantified by ImageJ. (**C**) The relative IFITM1 mRNA levels in the negative control (shCtrl) and ABHD17A knockdown (shABHD17A) HEK293 cells were determined via qRT-PCR. (**D**) Western blotting analysis of the endogenous IFITM1 in the shCtrl and shABHD17A HEK293 cells. (**E**) Schematic diagram of the APEGS assay. The S-palmitoylated cysteine sites were replaced with polyethylene glycol maleimide (mPEG-mal). (**F**) ABHD17A catalyzed the depalmitoylation of N-Ras and PSD-95. HEK293 cells were transfected with Flag-N-Ras or Flag-PSD-95 along with EGFP or ABHD17A-EGFP for 24 h. Cell lysates were subjected to APEGS with 1 M NEM, 1 M NH2OH, and 1 mM mPEG-Mal, and subsequently separated by SDS-PAGE and analyzed by Western blotting. PEGylation events are indicated. (**G**) The gray value of palmitoylated bands to input bands was calculated as the palmitoylated level. (**H**) HEK293 cells were cotransfected with the indicated expression plasmids, and 24 h later, cells were harvested and lysed. Cell lysates were subjected to the APEGS. (**I**) The gray value of palmitoylated IFITM1 bands to input IFITM1 bands was calculated as the palmitoylated level. (**J**) The S-palmitoylation modification level of IFITM1 in shCtrl and ABHD17A knockdown HEK293 cells was checked by APEGS and Western blotting. (**K**) The gray value of palmitoylated IFITM1 bands to input IFITM1 bands was calculated as the palmitoylated level. ns, not significant; *** *p* < 0.001; ** *p* < 0.01 (unpaired two-tailed *t* test).

To gain a comprehensive understanding of whether ABHD17A regulated the expression or post-translational modification (e.g., S-palmitoylation) of IFITM1, we, respectively, analyzed the mRNA and protein amounts of endogenous IFITM1 in ABHD17A overexpression and knockdown HEK293 cells. In neither case did ABHD17A affect the expression of IFITM1 (Figure 2A–D). As a depalmitoylating enzyme, ABHD17A has been implicated in the palmitate turnover on N-Ras and PSD-95 [17,21,22]. Here, by using Acyl-PEGyl exchange gel shift (APEGS) system [28], we witnessed the S-palmitoylated band of N-Ras and PSD-95 (Figure 2F). Overexpression of ABHD17A significantly decreased the S-palmitoylation modification of N-Ras and PSD-95 (Figure 2F,G), which are consistent with previous reports and validate the feasibility of the APEGS assay.

To investigate the necessity of ABHD17A in the palmitoylation/depalmitoylation cycle of IFITM1, we cotransfected Flag-IFITM1 with ABHD17A-EGFP in HEK293 and A549 cells, and subsequently cell lysates were subjected to the APEGS assay. Intriguingly, the S-palmitoylation modification level of IFITM1 was notably higher in ABHD17A overexpressing cells (Figure 2H,I and Figure S1B). In addition, we constructed a knockdown of ABHD17A and found that silencing ABHD17A expression reduced S-palmitoylated IFITM1 amounts when compared with scrambled control (Figure 2J,K). Altogether, the above results revealed an unconventional function of ABHD17A, that is, ABHD17A did not catalyze the depalmitoylation of IFITM1, but instead facilitated the palmitoylation modification of IFITM1.

### 3.3. The zDHHC Family Members Are Dispensable for ABHD17A-Mediated S-Palmitoylation of IFITM1

**Figure 3:**
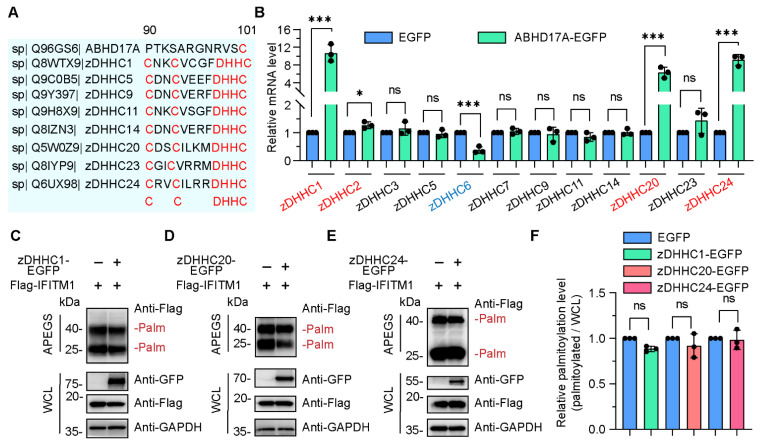
zDHHC members are dispensable for ABHD17A-mediated S-palmitoylation of IFITM1. (**A**) Conservation analysis of the amino acid sequence between ABHD17A and zDHHC family proteins. Multiple alignments of human ABHD17A with seven zDHHC members using the Uniprot website. The conserved residues and motif were marked in red color. (**B**) A small-scale screen was performed to identify candidate zDHHC members whose expression potentially changes in response to ABHD17A overexpression. The mRNA level of the individual zDHHC genes was verified by qRT-PCR in control and ABHD17A-EGFP-expressing cells. The upregulated and downregulated genes were indicated in red and blue fonts, respectively. ns, not significant; *** *p* < 0.001; * *p* < 0.05 (unpaired two-tailed *t* test). (**C**–**E**) APEGS assay for zDHHC members catalyzing the S-palmitoylation of IFITM1. HEK293 cells were cotransfected with expression plasmids encoding EGFP-tagged-zDHHC1 (**C**), zDHHC20 (**D**), zDHHC24 (**E**) along with Flag-IFITM1, and 24 h later, cell lysates were subjected to the APEGS process. (**F**) The gray value of palmitoylated IFITM1 bands to input IFITM1 bands depicted in (**C**–**E**) were calculated as the palmitoylated level. ns, not significant (unpaired two-tailed *t* test).

As a well-known depalmitoylase, how did ABHD17A upregulate the S-palmitoylation modification of IFITM1? One possibility is that ABHD17A functions as a palmitoylase, and the other is that ABHD17A participates in the dynamic S-palmitoylating modification of IFITM1 through intermediate regulatory factors. S-Palmitoylation modification was catalyzed by zDHHC family members [30]. The zDHHC members contain the conserved DHHC motif, which is indispensable for zDHHC acylation and enzymatic catalytic activity [31]. Conservation analysis of the amino acid sequence between ABHD17A and zDHHC members revealed that ABHD17A did not contain the conserved DHHC motif (Figure 3A), suggesting ABHD17A may not have the capacity to catalyze S-palmitoylation modification.

Previously, we and others have, respectively, reported that zDHHC3 and zDHHC20 enhanced the S-palmitoylation of IFITM1 and IFITM3 [6,12]. Thus, we sought to investigate whether ABHD17A increased the S-palmitoylation level of IFITM1 via zDHHC members. A limited zDHHC family screen was carried out, and the expression of zDHHC1, zDHHC20, and zDHHC24 were significantly upregulated in response to ABHD17A overexpression (Figure 3B). We speculated that ABHD17A may promote the S-palmitoylation modification of IFITM1 by upregulating these zDHHC proteins. However, overexpression of each of these three zDHHC proteins did not significantly affect the S-palmitoylation level of IFITM1 (Figure 3C–F). To sum up, these data indicated zDHHC family may not be necessary for ABHD17A upregulating the S-palmitoylation of IFITM1.

### 3.4. ABHD17A Promotes the S-Palmitoyl Modification Level of IFITM1 by Downregulating ABHD16A

**Figure 4:**
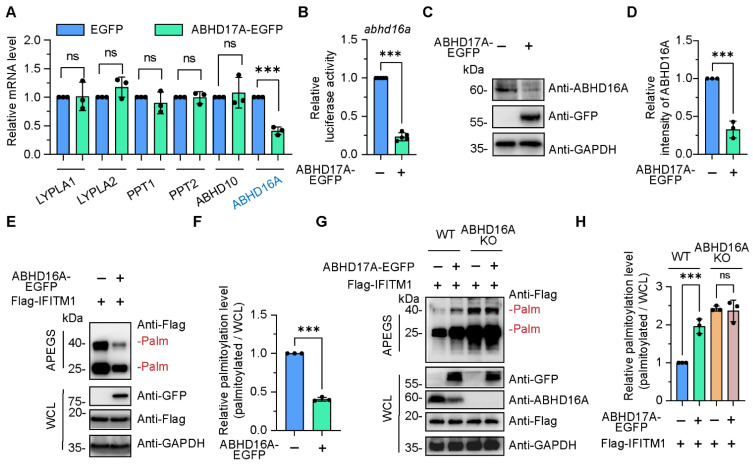
ABHD17A promotes the S-palmitoyl modification level of IFITM1 by inhibiting the expression of ABHD16A. (**A**) A small-scale screen was conducted to identify candidate depalmitoylases whose expression potentially changes upon ABHD17A overexpression. The mRNA level of the individual depalmitoylase genes was verified by qRT-PCR in control and ABHD17A-EGFP-expressing cells. The downregulated gene was indicated in blue font. (**B**) ABHD17A overexpression inhibited the activity of promoter of ABHD16A. HEK293 cells were cotransfected with pEGFP-N1 or pABHD17A-EGFP along with pGL3-ABHD16A-promoter, and 24 h later, the promoter of ABHD16A activity was checked via luciferase reporter assay. (**C**) Endogenous ABHD16A was downregulated by the expression of ABHD17A. The extracts of HEK293 cells with EGFP or ABHD17A-EGFP expression were subjected to Western blotting analysis. (**D**) Quantification of the protein levels of ABHD16A depicted in (**C**). (**E**) APEGS assay for ABHD16A catalyzing the depalmitoylation of IFITM1. HEK293 cells were cotransfected with expression plasmids of EGFP or EGFP-tagged ABHD16A along with Flag-IFITM1, and 24 h later, cell lysates were subjected to APEGS. (**F**) The gray value of palmitoylated IFITM1 bands to input IFITM1 bands according to the experimental design in (**E**) were calculated as the palmitoylated level. (**G**) ABHD16A plays an indispensable role in upregulating the S-palmitoylation modification level of IFITM1 by ABHD17A. CRISPR/Cas9 was used to knock out ABHD16A, and the knockout efficiency was verified by Western blotting with an endogenous ABHD16A antibody. (**H**) The gray value of palmitoylated IFITM1 bands to input IFITM1 bands according to the experimental design in (**G**) were calculated as the palmitoylated level. ns, not significant; *** *p* < 0.001; (unpaired two-tailed *t* test for (**A**,**B**,**D**,**F**); one-way ANOVA for (**H**)).

Apart from zDHHC members, the expression of enzymes that can catalyze the depalmitoylation reaction were also investigated in ABHD17A overexpression cells. These include acyl-protein thioesterases (LYPLA1 and LYPLA2) [14,15,32], palmitoyl-protein thioesterases (PPT1 and PPT2) [13], ABHD10 [18], and ABHD16A [4,5]. Among them, only the mRNA level of ABHD16A was remarkably downregulated upon ABHD17A overexpression (Figure 4A). Subsequently, the promoter activity and endogenous protein amount of ABHD16A were also checked in ABHD17A overexpression cells through luciferase reporter assay and immunoblotting, respectively. We found that ABHD17A inhibited the expression of ABHD16A (Figure 4B–D).

Previously, we revealed ABHD16A as a novel depalmitoylase that specifically interacts and downregulates IFITM1 in human, swine, and mouse cells (Figure 4E,F and Figure S2) [4,5]. We wondered whether the enhanced S-palmitoylation level of IFITM1 was due to the inhibition of ABHD16A by ABHD17A. We constructed ABHD16A-deficient cells via CRISPR/Cas9, and then employed the APEGS assay to assess the effect of ABHD17A overexpression on S-palmitoylation of IFITM1 in wild-type and ABHD16A knockout cells. It is worth noting that increased S-palmitoylated IFITM1 bands were observed in cells expressing ABHD17A-EGFP compared to EGFP-expressing cells, while ABHD17A no longer upregulated the S-palmitoylation modification of IFITM1 in the absence of ABHD16A (Figure 4G,H), suggesting that ABHD17A enhanced the S-palmitoyl modification level of IFITM1 by inhibiting the expression of ABHD16A.

Next, we sought to investigate whether changing ABHD17A levels has a general impact on other substrate’s palmitoylation level. We focused on mouse derived IFITM3 because our previous research showed that mouse ABHD16A (mABHD16A) only interacts with mIFITM3 instead of mIFITM1 and mIFITM2, and specifically deacetylates mIFITM3 [5]. As expected, overexpression of mABHD17A-EGFP led to enhanced S-palmitoylation level of mIFITM3 (Figure S3), which proves that the S-palmitoylation post-translational modifications of the IFITM members are regulated by ABHD17A-ABHD16A among different species.

### 3.5. The Activity of IFITM1 to Restrict Virus Infection Is Positively Regulated by ABHD17A

**Figure 5:**
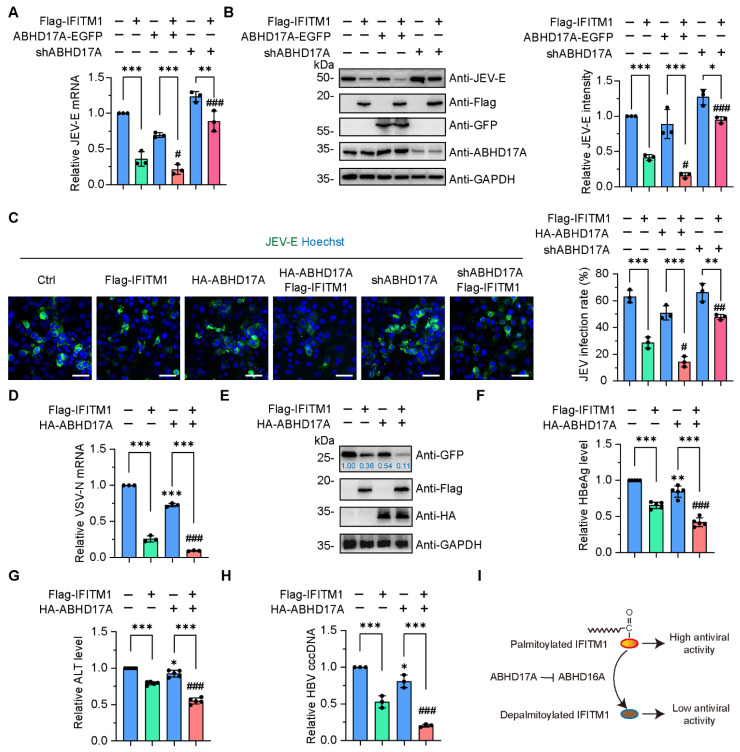
ABHD17A positively regulates the antiviral activity of IFITM1. (**A**) HEK293 cells were introduced with the indicated plasmids. After 24 h, the cells were infected with JEV (SA-14-14-2 strain) at an MOI of 0.1 for 24 h. The mRNA levels of JEV-E were detected by qRT-PCR. (**B**) The intracellular JEV-E protein amount was verified by Western blotting. (**C**) Transfected HEK293 cells were infected with JEV (SA-14-14-2 strain) at an MOI of 0.1 for 2 days. Cells were subsequently stained with JEV-E glycoprotein antibody (green) and Hoechst for the nucleus (blue), respectively, to detect the infection rates. Scale bar, 100 μm. The virus infection rate was calculated for each indicated groups. (**D**) HEK293 cells were transfected with the indicated plasmids. Then, 24 h later, cells were infected with VSVΔG pseudotypes at an MOI of 0.1. The mRNA levels of VSV-N were detected by qRT-PCR. (**E**) HEK293 cells were transfected with the indicated plasmids and then infected with VSVΔG pseudotypes (MOI: 0.1) for 24 h, and the GFP levels were analyzed using Western blotting. (**F**,**G**) HBeAg and ALT in the supernatant of HepG2 2.215 cells transfected with the indicated plasmids were calculated, respectively. (**H**) The HBV cccDNA in HepG2 2.215 cells transiently transfected with pcDNA3.1-Flag, Flag-IFITM1, ABHD17A-EGFP, and ABHD17A-EGFP; Flag-IFITM1 was analyzed using qRT-PCR, respectively. (**I**) Schematic diagram of ABHD17A facilitates the antiviral activity of IFITM1 by inhibiting the expression of depalmitoylase ABHD16A. ns, not significant; * *p* < 0.05; ** *p* < 0.01; *** *p* < 0.001 (wild-type cells as the Ctrl; one-way ANOVA). # *p* < 0.05; ## *p* < 0.01; ### *p* < 0.001 (Flag-IFITM1 overexpressing cells as the Ctrl, one-way ANOVA).

Previously, we reported that S-palmitoylation modification enables IFITM1 to be more localized on the plasma membrane, thereby resisting the invasion of various viruses, such as Japanese encephalitis virus (JEV), a flavivirus that causes epidemics of encephalitis in humans and reproductive disorders in swine [5,33]. To address whether ABHD17A affects the antiviral activity of IFITM1, transfected HEK293 or A549 cells were infected with JEV for 24 h. The epidemic encephalitis live vaccine (SA14-14-2) has a virus content of 1 × 10^5^ pfu, so in actual operation, we will infect 1 × 10^6^ cells with the 1 × 10^5^ pfu live vaccine to achieve a multiplicity of infection (MOI) of 0.1. Subsequently, we, respectively, measured the mRNA and protein amounts of JEV envelope protein (JEV-E). Overexpression of ABHD17A promoted IFITM1 in inhibiting JEV replication (Figure 5A,B and Figures S1C and S4). In contrast, the antiviral effects of IFITM1 were counteracted in response to ABHD17A silencing (Figure 5A,B and Figures S1C and S4). Furthermore, we calculated the percentage of JEV-positive cells by implementing the immunostaining with JEV-E antibody. JEV-E signals were detected in the cytoplasm, the presence of exogenous IFITM1 expression reduced the infection rate of JEV (Figure 5C). Overexpression of ABHD17A with IFITM1 enabled most cells to resist JEV infection, while the knockdown of ABHD17A counterbalanced the antiviral effects exerted by IFITM1 (Figure 5C).

Since IFITM1 has broad-spectrum antiviral functions, combined with our previous findings that ABHD16A negatively regulates the antiviral effect of IFITM1 against various viral infections [5,34] (Figure S5), we were curious whether ABHD17A could regulate IFITM1’s ability to resist other types of viral infections. The recombinant vesicular stomatitis virus (VSV) was a widely used pseudotyped virus for studying the mechanism of RNA virus–host interaction, in which the glycoprotein coding sequence has been replaced by a gene encoding GFP [35]. By detecting the transcription of the nucleocapsid protein (VSV-N) encoding gene and the protein expression level of free GFP, we witnessed that ABHD17A further enhanced the antiviral effect of IFITM1, resulting in a very weak replication ability of VSV pseudovirus in IFITM1 and ABHD17A coexpressing cells (Figure 5D,E). Recently, we and Ye et al. revealed that the replication of the DNA virus Hepatitis B virus (HBV) was substantially inhibited by IFITM1 [34,36]. We cotransfected the expression plasmids of Flag-IFITM1 with ABHD17A-EGFP in HepG2.215 cells with HBV replication, and confirmed ABHD17A facilitated IFITM1 restricting HBV replication by individually estimated the level of extracellular Hepatitis B e antigen (HBeAg), Hepatitis B surface antigen (HBsAg), and HBV DNA (Figure 5F–H). Taken together, these data revealed that ABHD17A promotes IFITM1 to restrict various virus infections.

## 4. Discussion

In the present study, we elucidated an unprecedented function of depalmitoylase ABHD17A in virus infection (Figure 5I). We demonstrated that (1) ABHD17A interacted with IFITM1 on the plasma membrane, ABHD17A did not remove the palmitate from IFITM1, instead, it contributes to the S-palmitoylation and antiviral activity of IFITM1. (2) The novel found depalmitoylase ABHD16A was apparently downregulated in response to ABHD17A overexpression, which contributes to the increased S-acylated amount of IFITM1. Together, our data identified two ABHD family members with opposite but related roles in regulating the dynamic modification of IFITM1 S-palmitoylation and expanded the novel function of ABHD17A in innate immunity.

ABHD17A has been reported to exert depalmitoylase activity for 10 years, it participates in multiple biological processes such as cancer growth, synapse development, and inflammation by removing palmitoyl groups from substrates [21,22,24,25,37]. However, it is still unknown whether ABHD17A is involved in the virus–host interaction. Interferons are indispensable cytokines that arouse the expression of interferon-stimulated genes (ISGs) among vertebrates [38,39,40]. The ISG-encoded IFITMs are localized on the cell membrane to resist the invasion of various viruses, which is regulated by S-palmitoylation modification [12,41]. However, apart from our discovery of ABHD16A [4,5,34], there are currently no reports of enzymes that specifically mediate the S-palmitoylation of IFITM1. In the current study, we witnessed an unexpected effect that ABHD17A facilitated the S-acylation of IFITM1, which enhanced the antiviral capability.

How does ABHD17A, a classic depalmitoylase, enhance the S-palmitoylation modification of IFITM1? We analyzed the distinct subcellular distribution of depalmitoylases in ABHD members. Cao et al. indicated ABHD10 mediated the depalmitoylation reaction on mitochondria [18]. ABHD16A has been reported to be more localized on the intracellular endoplasmic reticulum (ER) membrane [42]. Consistently, we previously reported ABHD16A shifted IFITM1 away from the plasma membrane to endosome membranes to achieve the depalmitoylation reaction [5]. In contrast, ABHD17A has been identified to be more localized on plasma membrane in this work and according to previous studies [13,21]. Several groups have demonstrated that the N-terminus of ABHD17A is rich in conserved cysteine residues [13,21,43], which allows ABHD17A to undergo itself palmitoylation modification. Palmitoylated ABHD17A is localized on the plasma membrane, which facilitates its proximity to substrates [13]. Therefore, different subcellular localizations may determine the members of ABHD family exert diverse effects on IFITM1.

Given that the zDHHC members normally undergo autopalmitoylation on the DHHC cysteine residues, and subsequently transfer the palmitate group to substrate proteins [30,44], we wondered whether the self-palmitoylated ABHD17A achieved by N-terminal cysteines has the potential activity of palmitoyl acyltransferases. To address this, we aligned the sequence of human ABHD17A with zDHHC members, and found ABHD17A lacks the DHHC motif, which plays an indispensable role in palmitate transfer. Combined with the N-terminal cysteine-rich domain of ABHD17A was not necessary for enzyme activity [13], we assumed that ABHD17A could not be a palmitoylating enzyme; ABHD17A may regulate the S-acylation of IFITM1 via other palmitoylating/depalmitoylating enzymes. Three zDHHCs (zDHHC1, zDHHC20, and zDHHC24) were upregulated upon ABHD17A overexpression. However, none of them can catalyze the S-acylation of IFITM1. These upregulated zDHHCs may be induced by the host cell to balance the increased depalmitoylation reaction caused by excessive expression of ABHD17A. In addition, zDHHC3, which is responsible for IFITM1 S-palmitoylation in swine cells [6], was not altered in response to ABHD17A overexpression in HEK293 cells. Further research is needed to reveal which zDHHC is necessary for the S-palmitoylation modification of IFITM1, as well as to reveal the regulatory relationship and significance between ABHD17A and zDHHC family members.

In addition to zDHHCs, we found the expression of depalmitoylase ABHD16A was notably downregulated in ABHD17A overexpressing cells. Further study illustrates that the promoter activity of ABHD16A was inhibited upon ABHD17A expression. Since we did not observe the localization of ABHD17A in the cell nucleus, we wondered how ABHD17A affects the transcription of ABHD16A. We predicted the transcription factor of ABHD16A via UCSC Genome Browser, the CCAAT/enhancer binding protein β (C/EBP-β) obtained the highest score. Moreover, Yang et al. reported that the transcription of ABHD16A was regulated by MiR-4646-5p, a mirtronic miRNA, which derived from the splicing product of intron-3 within the genomic sequence of ABHD16A [45]. Subsequent work needs to clarify whether ABHD17A affects the expression of ABHD16A through the aforementioned transcription factors or miRNA. In addition, we queried the human-derived ABHD17A interaction network through two resources: The Molecular INTeraction Database (MINT, https://mint.bio.uniroma2.it/, accessed on 15 January 2012) and the protein interaction library using Next-generation sequencing analysis [46,47]. Fourteen proteins were found that interacted with ABHD17A, of which six were related to transcription factors. They are TNF receptor-associated factor 1 (TRAF1), TNFAIP3-interacting protein 1 (TNIP1), MyoD family inhibitor (MDFI), Histone-lysine N-methyltransferase EHMT2 (EHMT2), Zinc finger protein RFP (TRIM27), and Baculoviral IAP repeat-containing protein 2 (BIRC2). Both EHMT2 and TRIM27 have been reported to be able to negatively regulate transcription process [48,49]. Moreover, through SwissPalm (https://swisspalm.org/) website analysis, we found that TRIM27 is the protein most likely to undergo S-palmitoylation post-translational modification among all 14 proteins that interact with ABHD17A. Therefore, ABHD17A may regulate transcription factor activity through palmitoylation modification, thereby mediating the expression of proteins such as ABHD16A. Subsequent work needs to be gradually explored and verified.

Recently, we discovered that ABHD16A, the only found depalmitoylase targeting IFITM1, was ubiquitinated and degraded by E3 ubiquitin ligase ring finger protein 5 (RNF5) in swine cells [4]. Combined with the finding that ABHD17A inhibits ABHD16A expression discovered in this work, we extended the upstream molecular mechanism of ABHD16A, and proposed a potential dynamic cycle in which ABHD16A and ABHD17A balance the IFITM1 S-palmitoylation modification.

Growing evidence has indicated that S-palmitoylation regulates both innate immunity and viral protein activity [29,50,51]. When host cells are infected with DNA viruses, S-palmitoylation modification of stimulator of interferon genes (STING) facilitates the expression and release of IFNs and proinflammatory cytokines [52,53]. Furthermore, in the canonical IFN signaling pathways, the palmitoylation modification of IFN receptor IFNAR1, janus kinase/signal transducer and activator of transcription (JAK/STAT) were successively investigated [15,54,55]. IFITM1 was induced by IFNs and the JAK/STAT pathway. In the current study, we first verified that the transcription and expression of IFITM1 were not altered upon ABHD17A overexpression, which indicated that the signaling pathways upstream of IFITM1 may not be affected by ABHD17A expression. Furthermore, S-palmitoylation of viral proteins was also important for viral replication and virulence. The cysteines in the nonstructural protein 2A (NS2A) of JEV and the transmembrane glycoprotein (G protein) of VSV were required for palmitate addition [56,57]. It is thus tempting to speculate that ABHD17A may play multiple roles in virus–host interaction through catalyzing the depalmitoylation reaction or increasing S-palmitoylation by inhibiting ABHD16A.

## 5. Conclusions

In summary, we have characterized the unique role of ABHD17A in increasing the S-palmitoylation and antiviral activity of IFITM1. The well-known depalmitoylase ABHD17A interacted with IFITM1, but did not initiate the palmitate-removing reaction. Mechanistically, the knockdown of ABHD17A suppressed the S-palmitoylation modification on IFITM1. We further demonstrated that ABHD17A downregulated the expression of ABHD16A, which reduced the depalmitoylation effect on IFITM1, and consequently enhanced the antiviral activity of IFITM1. Altogether, our study fills the gap in our knowledge of the cellular function of ABHD17A in viral infection and provides new insight into the mechanism of ABHD members in the dynamic S-palmitoylation of IFITM protein.

## Data Availability

The original contributions presented in this study are included in the article/Supplementary Materials. Further inquiries can be directed to the corresponding author.

## Supplementary Materials

The following supporting information can be downloaded at: https://www.mdpi.com/article/10.3390/biom15070992/s1, Figure S1: ABHD17A interacts with IFITM1 and increases the S-palmitoylated and antiviral effects of IFITM1 in A549 cells. Figure S2: ABHD16A interacts with IFITM1 and catalyzes the S-depalmitoylation of IFITM1. Figure S3: Mouse ABHD17A increased the S-palmitoylation level of mouse IFITM3. Figure S4: ABHD17A positively regulates the antiviral activity of IFITM1. Figure S5: ABHD16A negatively regulates the antiviral function of IFITM1. Table S1: The antibody used in this study. Table S2: The primer for qRT-PCR experiments used in this study. Original images for Blots.

## Abbreviations

The following abbreviations are used in this manuscript:

ABHD	α/β-hydrolase domain
IFITMs	interferon-inducible transmembrane proteins
zDHHC	zinc finger Asp–His–His–Cys domain
PTM	post-translational modification
APEGS	acyl-PEGyl exchange gel shift
JEV	Japanese encephalitis virus
VSV	vesicular stomatitis virus
HBV	hepatitis B virus
