# Supplementary material for: The Unconventional Role of ABHD17A in Increasing the S-Palmitoylation and Antiviral Activity of IFITM1 by Downregulating ABHD16A
Source: Biomolecules. 2025 Jul 11;15(7):992. doi: 10.3390/biom15070992 (PMC12292810; doi:10.3390/biom15070992)
Supplement: Supplementary file 1 [file biomolecules-15-00992-s001.zip › biomolecules-3683916-supplementary.pdf]

Supplementary File

Figure S1: ABHD17A interacts with IFITM1 and increases the S-palmitoylated and antiviral effects of IFITM1 in A549 cells.

Figure S2: ABHD16A interacts with IFITM1 and catalyzes the S-depalmitoylation of IFITM1.

Figure S3: Mouse ABHD17A increased the S-palmitoylation level of mouse IFITM3.

Figure S4: ABHD17A positively regulates the antiviral activity of IFITM1.

Figure S5: ABHD16A negatively regulates the antiviral function of IFITM1.

Table S1: The antibody used in this study.

Table S2: The primer for qRT-PCR experiments used in this study.

Original Images for Blots

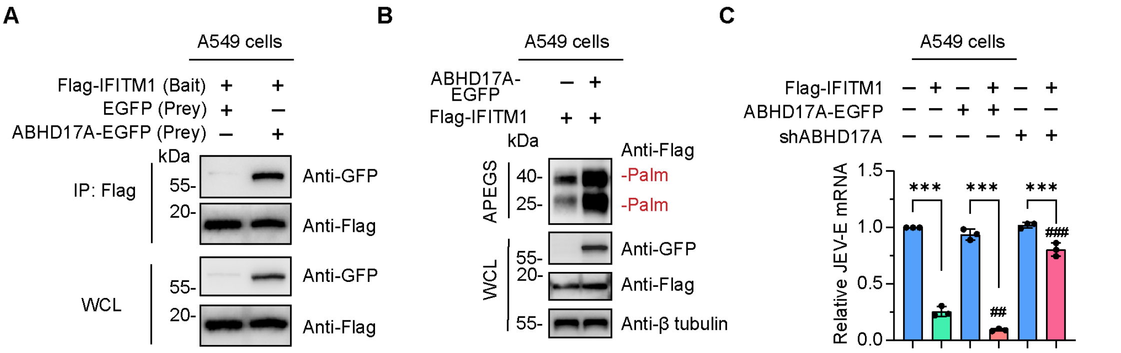

Figure S1. ABHD17A interacts with IFITM1 and increases the S-palmitoylated and antiviral effects of IFITM1 in A549 cells. (**A**) A549 cells were transfected with expression plasmids of EGFP-N1 or ABHD17A-EGFP along with a plasmid encoding Flag-IFITM1. 24 h post-transfection, immunoprecipitation was performed with the Protein A+G magnetic beads binding with Flag antibody, and the whole-cell lysates (WCL) and IPs were analyzed by Western blotting. (**B**) A549 cells were cotransfected with the indicated expression plasmids, and 24 h later, cells were harvested and lysed.

Cell lysates were subjected to the APEGS process and analyzed by Western blotting. (**C**) A549 cells were transfected with the indicated plasmids. 24 h later, cells were infected with JEV (SA-14-14-2 strain) at an MOI of 0.1 for 24 h. The mRNA levels of JEV-E were detected by qRT-PCR. Statistical analysis data are mean±SD from three independent experiments. ns, not significant; **P* < 0.05; ***P* < 0.01; ****P* < 0.001 (wild-type cells as the Ctrl; one-way ANOVA). # *P* < 0.05; ## *P* < 0.01; ### *P* < 0.001 (single transfection of Flag-IFITM1 as the Ctrl, one-way ANOVA).

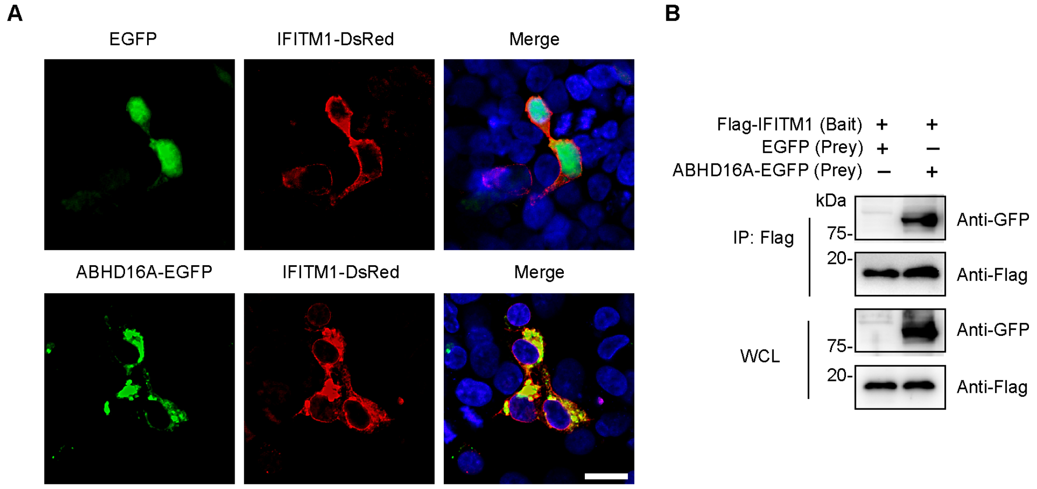

Figure S2. ABHD16A interacts with IFITM1 and catalyzes the S-depalmitoylation of IFITM1. (**A**) Representative confocal images showing ABHD16A colocalized with IFITM1. HEK293 cells were cotransfected with expression plasmids of IFITM1-DsRed with EGFP or ABHD16A-EGFP, respectively. 24 hours later, cells were fixed with 4% paraformaldehyde, and nuclei were stained with Hoechst 33342. Scale Bar, 20 μm. (**B**) ABHD16A interacts with IFITM1. HEK293 cells were transfected with expression plasmids encoding EGFP or ABHD16A-EGFP along with a plasmid encoding Flag-IFITM1. 24 h post-transfection, immunoprecipitation was performed with the Protein A+G magnetic beads binding with Flag antibody, and the whole-cell lysate (WCL) and IP were analyzed by Western blotting.

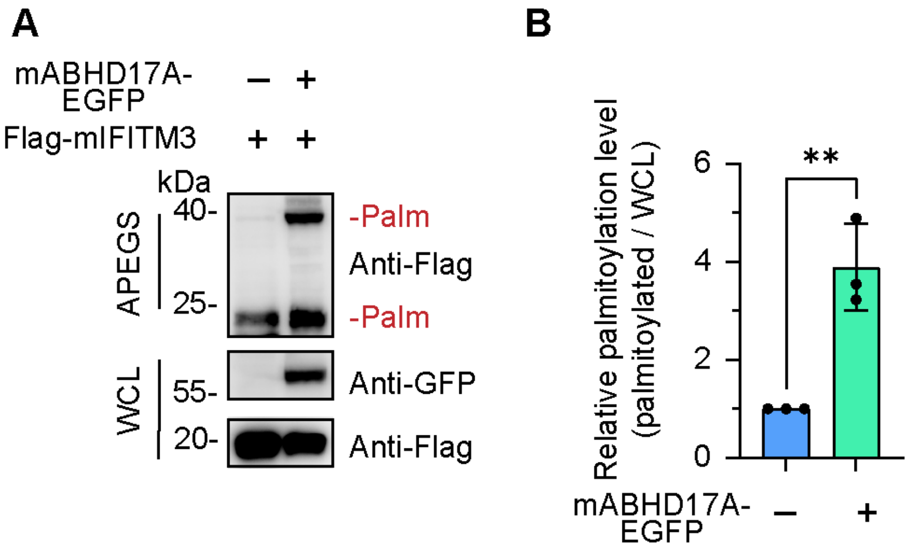

Figure S3. Mouse ABHD17A increased the S-palmitoylation level of mouse IFITM3. (**A**) NIH/3T3 cells were cotransfected with the indicated expression plasmids, and 24 h later, cells were harvested and lysed. Cell lysates were subjected to the APEGS. (**B**) The gray value of S-palmitoylated mIFITM3 bands to input mIFITM3 bands was calculated as the palmitoylated level. ns, not significant; ***P* < 0.01 (unpaired two-tailed t test). Experiments were repeated independently three times.

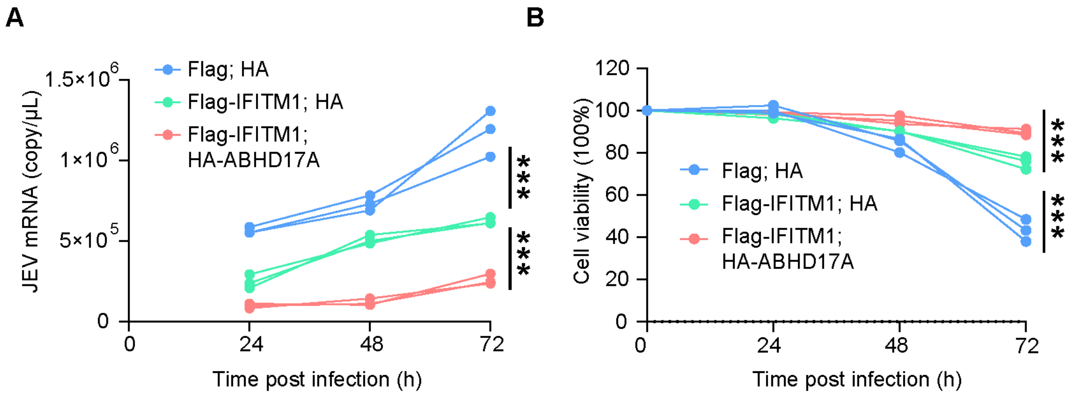

Figure S4. ABHD17A positively regulates the antiviral activity of IFITM1. (**A**) Replication kinetics of extracellular JEV in the indicated groups. (**B**) Quantification of the cell viability in the indicated groups. Experiments were repeated independently three times. ns. not significant; *P* < 0.05; ***P* < 0.01; ****P* < 0.001 (one-way ANOVA).

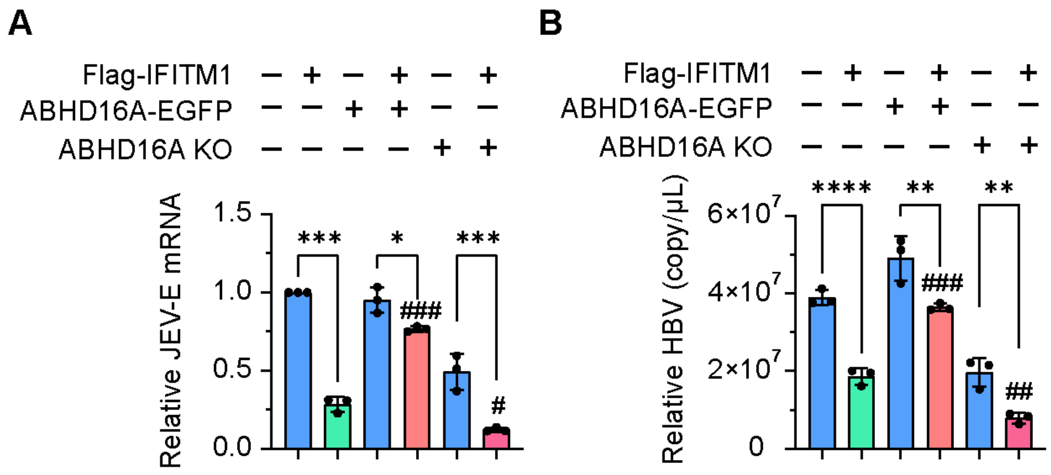

Figure S5. ABHD16A negatively regulates the antiviral function of IFITM1. (**A**) Wild-type and ABHD16A KO HEK293 cells were introduced with the indicated plasmids. 24 h later, cells were infected with JEV (SA-14-14-2 strain) at an MOI of 0.1 for 24 h. The mRNA levels of JEV-E were detected by qRT-PCR. (**B**) The HBV DNA in the extracellular media of wild-type and ABHD16A KO HepG2.215 cells transiently transfected with indicated expression plasmids were analyzed by qRT-PCR. Experiments were repeated independently three times. ns. not significant; *P < 0.05; **P < 0.01; ***P < 0.001. #P < 0.05; ##P < 0.01; ###P < 0.001 (Flag-IFITM1 overexpressing cells as the Ctrl, one-way ANOVA).

Table S1. Antibodies used in this study

Antibody	Host	Manufacturer	Cat No.
ABHD17A	Rabbit	Proteintech	15854-1-AP
IFITM1	Mouse	Proteintech	60074-1-Ig
ABHD16A	Rabbit	Saier Biotech	SRP08788
JEV-E	Mouse	Abcam	ab41671
GFP	Mouse	Proteintech	66002-1-Ig
Flag	Mouse	Sigma-Aldrich	F3165
HA	Rabbit	Proteintech	51064-2-AP
GAPDH	Rabbit	Proteintech	10494-1-AP
Beta Tubulin	Mouse	Proteintech	66240-1-Ig

Table S2. The primer for qRT-PCR experiments used in this study

Gene type	Gene name	Forward primer 5′ to 3′	Reverse primer 5′ to 3′
Endogenous Gene	IFITM1	atcctgttactggtattcgg	tataaactgctgtatctagg
Endogenous Gene	zDHHC1	gccgtctccatcgatccagc	tagttccgctcgcccacac
Endogenous Gene	zDHHC2	tgaattctgtcagacccttg	cgcctaagaacttcctgatg
Endogenous Gene	zDHHC3	ctggtttctggtcctctatg	ggcatttcctttgggcactg
Endogenous Gene	zDHHC5	cagccgccatcttcctag	ccttgtcctcatcctcctcag
Endogenous Gene	zDHHC6	ggttatcaagtttgaaaatc	agtaattataaagaatcatg
Endogenous Gene	zDHHC7	ctcttcatcgtcctcctcctc	ccgttgaccacagagtacc
Endogenous Gene	zDHHC9	cagtgaccctggagtgattc	ggtcgaagcgctccacacag
Endogenous Gene	zDHHC11	tgctcatcttccacatctacc	ggcgtgagttcagcttcttg
Endogenous Gene	zDHHC14	tacctggcggtgaaaatcac	tccagatcggcggcttcatc
Endogenous Gene	zDHHC20	tgctcagcctgtgactcatg	cgtgtatctgtcagttcattcg
Endogenous Gene	zDHHC23	atcggaatggggaaaagaac	tgaaggaagacaggcagcag
Endogenous Gene	zDHHC24	cctgtgcctgctgcttcatg	actcctctttgtggtggtcag
Endogenous Gene	LYPLA1	tactgcagtccacaagaatc	ttgcctcagggctgttctgcg
Endogenous Gene	LYPLA2	tggagccgtgtggtgtatgtg	cacagggatcctaggcgcatg
Endogenous Gene	PPT1	gcctgtggctcttggctgtgg	ggagtacgccccagcattcagt
Endogenous Gene	PPT2	gcggcgtgggtcctgcttctg	tgttcccacaggggtcgcaag

Endogenous Gene	ABHD10	ttgcgcgcttggcagctgtg	tggtcgattaaggaatgagag
Endogenous Gene	ABHD16A	gatacgtactatcagccccgtg	aggcgaagggagaggagtaat
Envelope protein gene of JEV	JEV-E	actgacatctcgacggtggc	ctcccaatcgctttactggt
Nucleocapsid protein gene of VSV	VSV-N	gatagtaccggaggattgacgacta	tcaaaccatccgagccattc
HBV DNA	HBV DNA	gttgcccgtttgtcctctaattc	ggagggatacatagaggttcctt
Reference Gene	GAPDH	gaccacagtccatgccatcac	gcctgcttcaccaccttctt

Raw image files of Western blot or gels

Fig. 1D

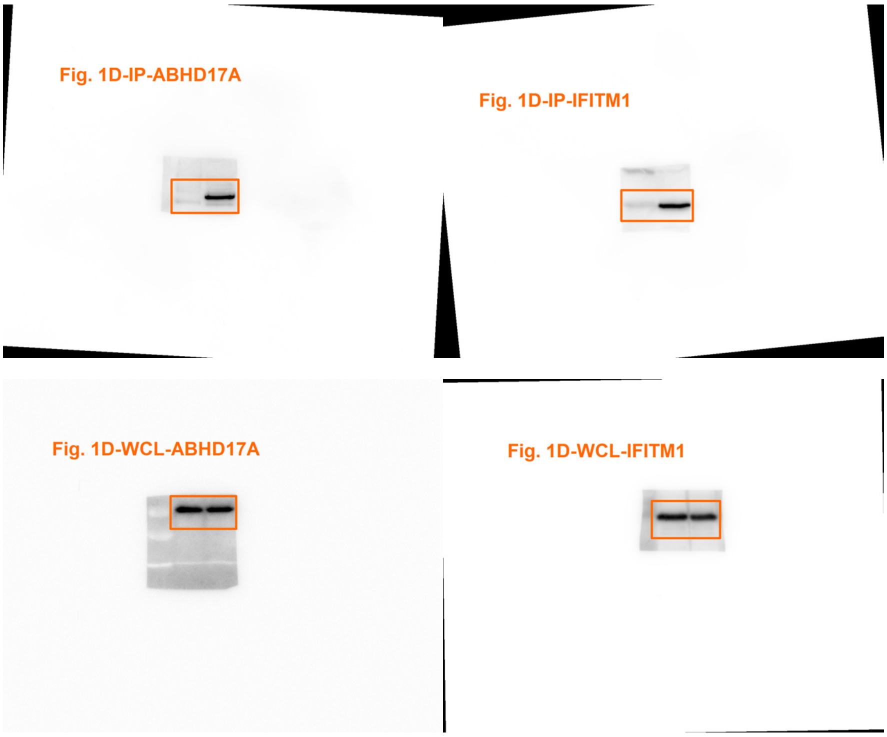

Fig. 1E

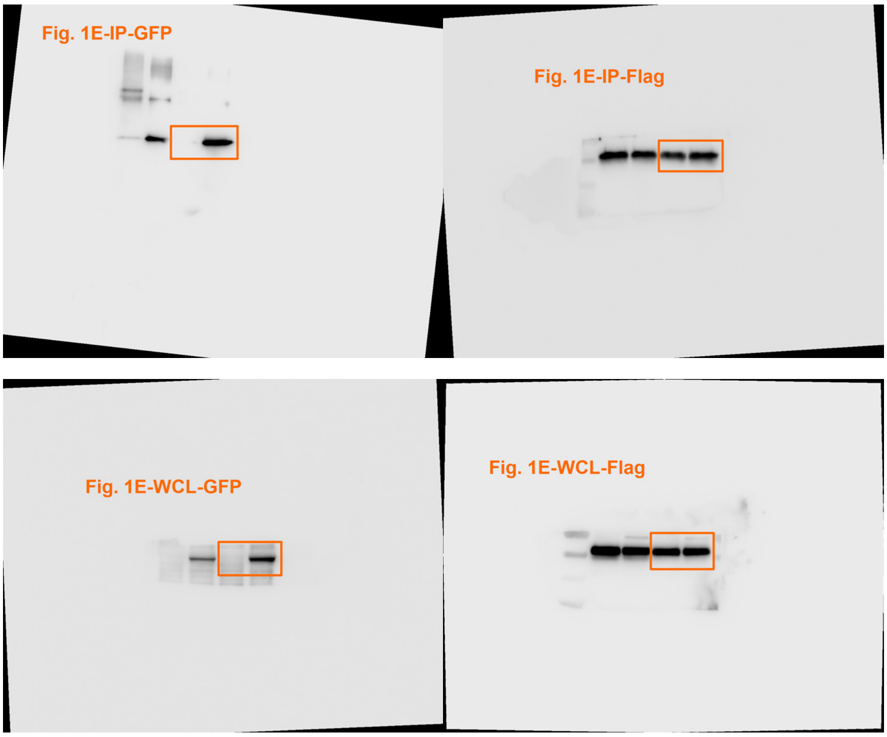

Fig.1F

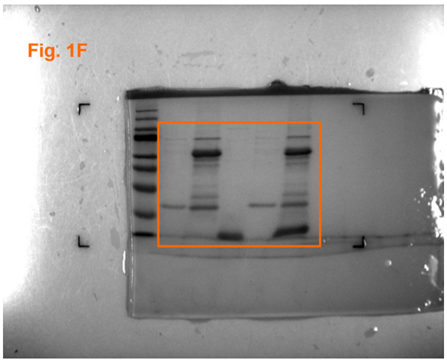
Fig. 1F

Fig. 2B

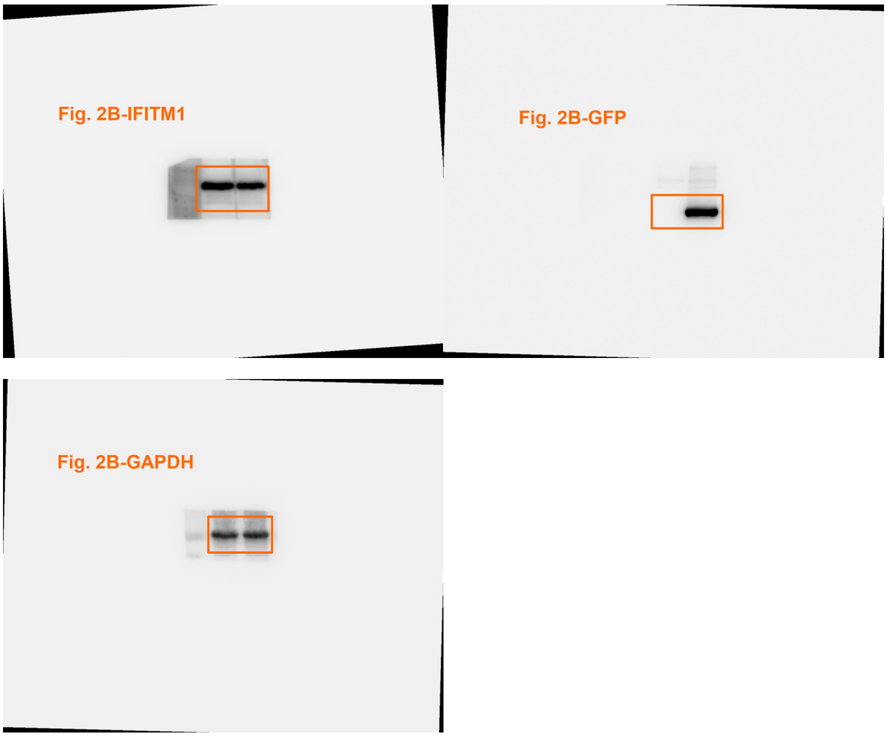
Fig. 2B-IFITM1
Fig. 2B-GFP
Fig. 2B-GAPDH

Fig. 2D

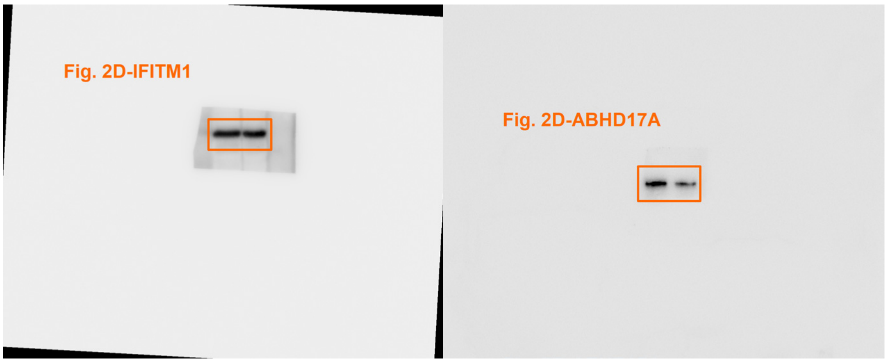
Fig. 2D-IFITM1
Fig. 2D-ABHD17A

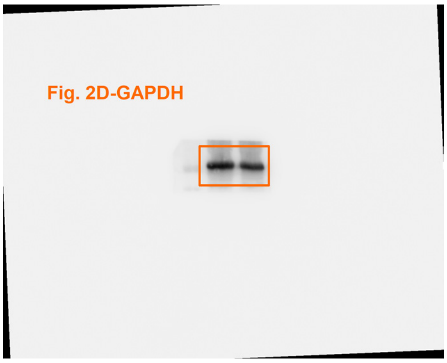

Fig. 2F-N-Ras

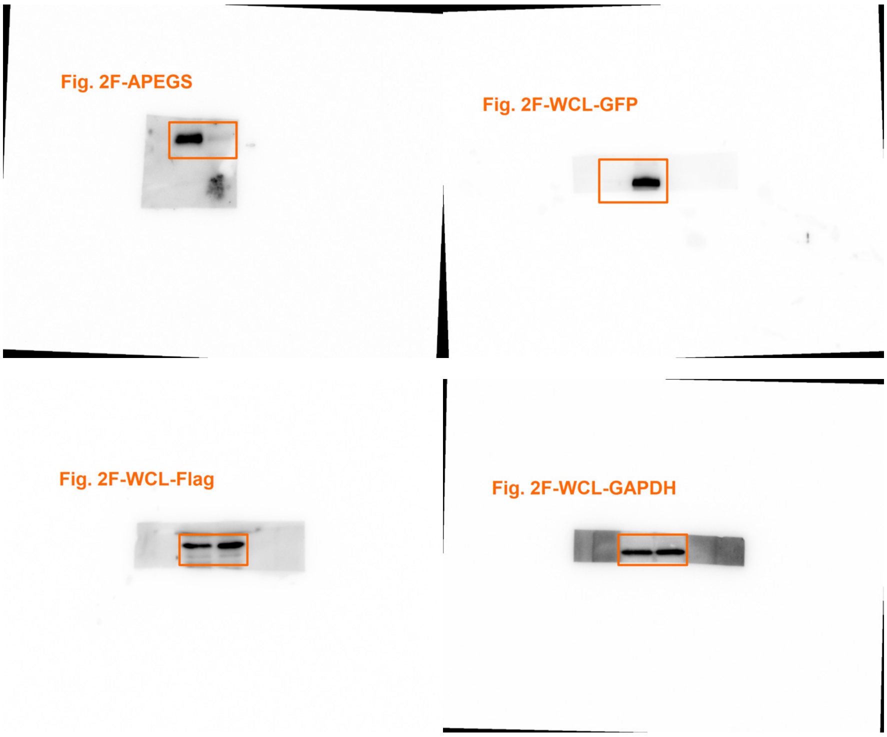

Fig. 2F-PSD-95

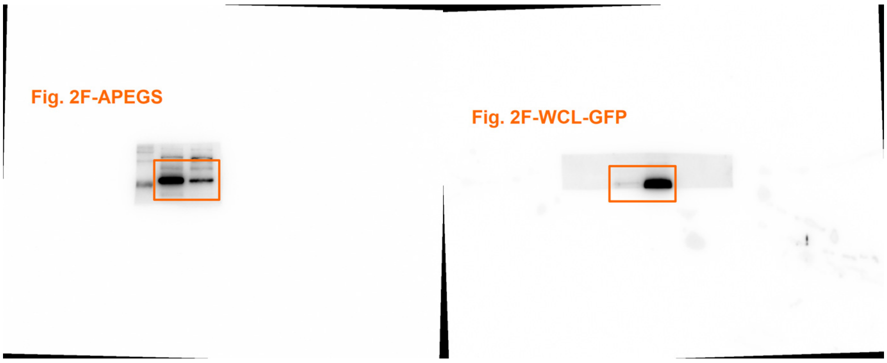

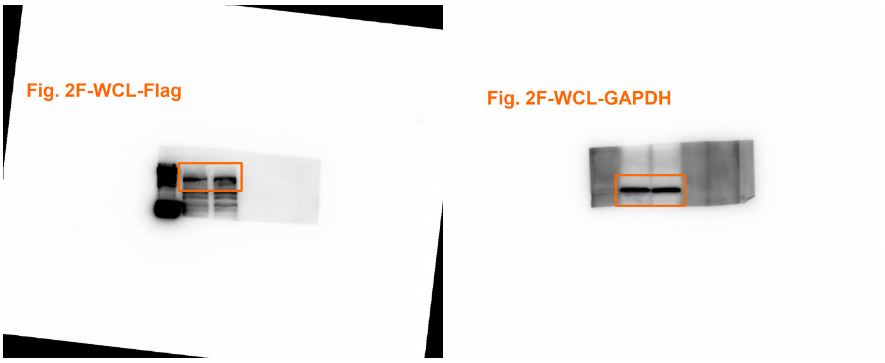
Fig. 2F-WCL-Flag
Fig. 2F-WCL-GAPDH

Fig. 2H

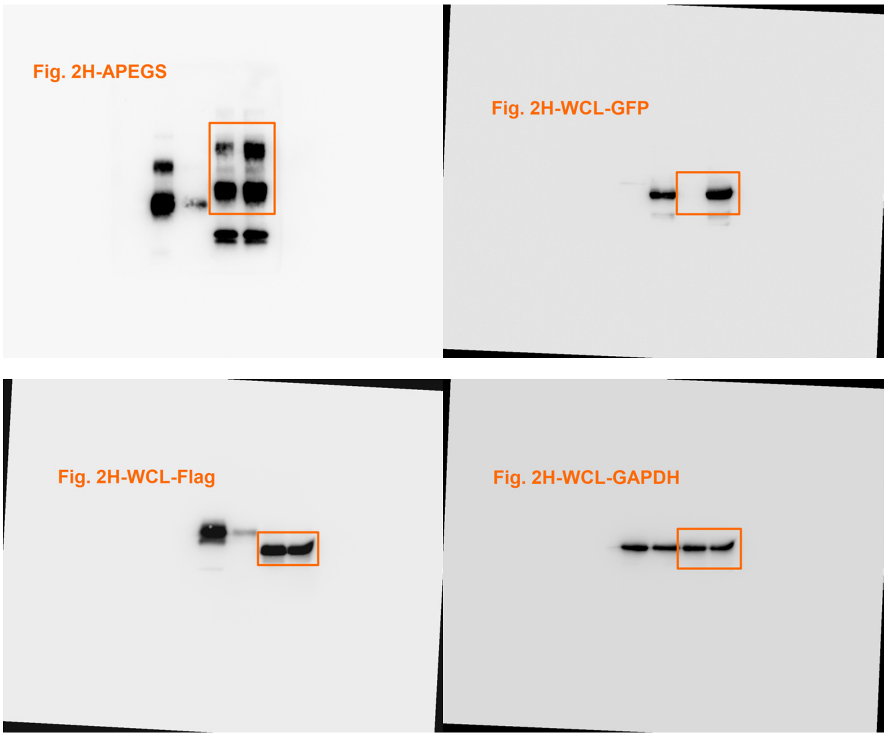
Fig. 2H-APEGS
Fig. 2H-WCL-GFP
Fig. 2H-WCL-Flag
Fig. 2H-WCL-GAPDH

Fig. 2J

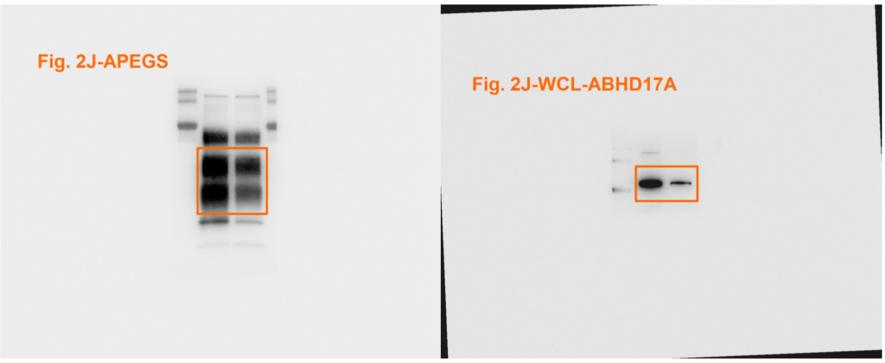
Fig. 2J-APEGS
Fig. 2J-WCL-ABHD17A

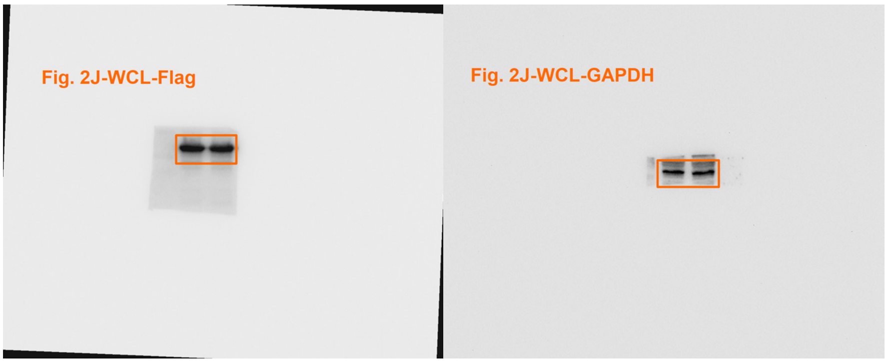
Fig. 2J-WCL-Flag
Fig. 2J-WCL-GAPDH

Fig. 3C

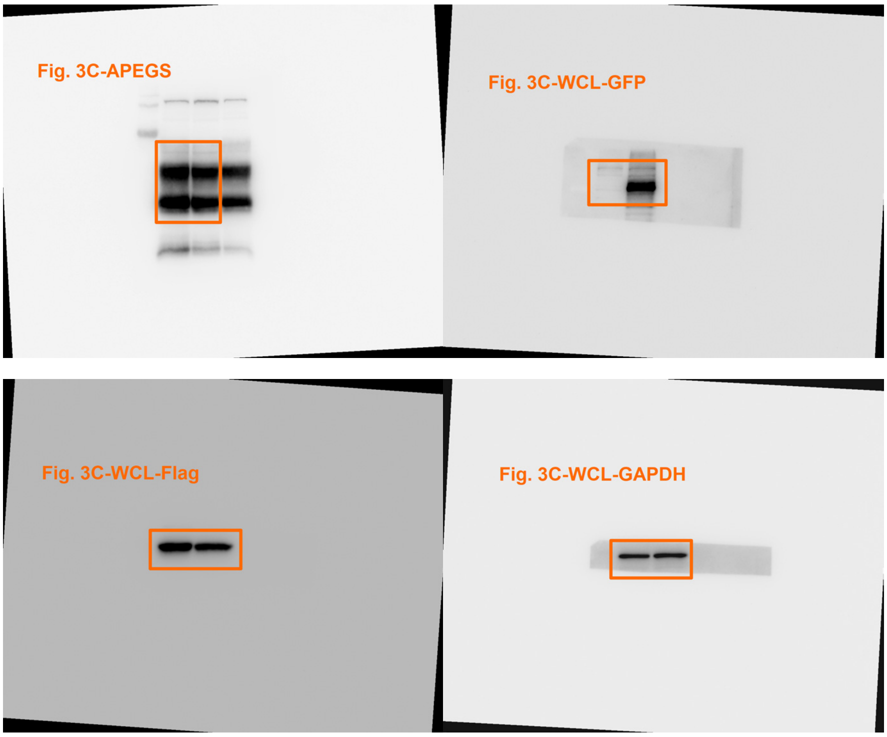
Fig. 3C-APEGS
Fig. 3C-WCL-GFP
Fig. 3C-WCL-Flag
Fig. 3C-WCL-GAPDH

Fig. 3D

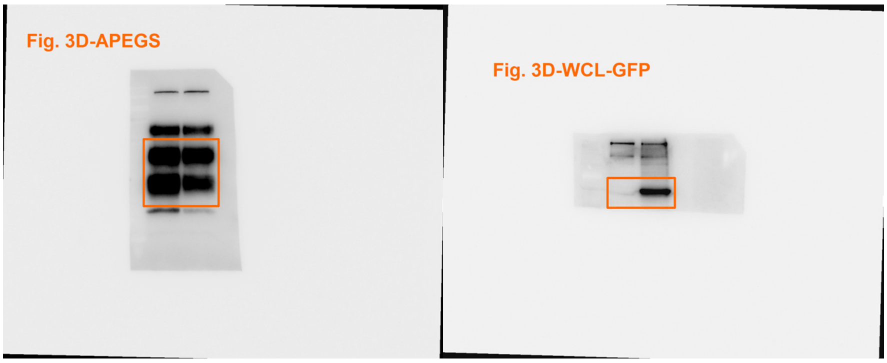
Fig. 3D-APEGS
Fig. 3D-WCL-GFP

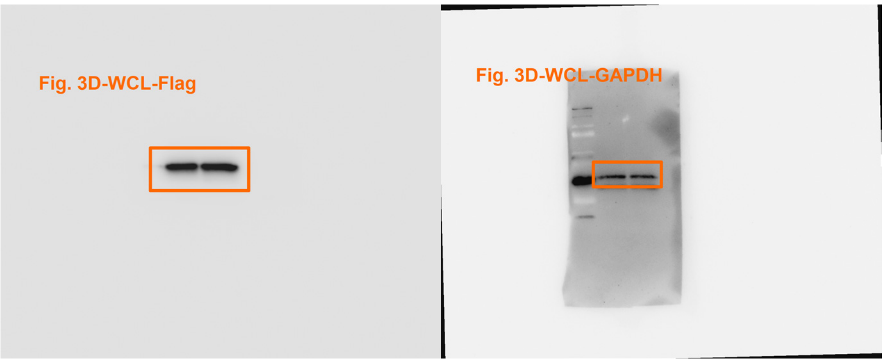
Fig. 3D-WCL-Flag
Fig. 3D-WCL-GAPDH

Fig. 3E

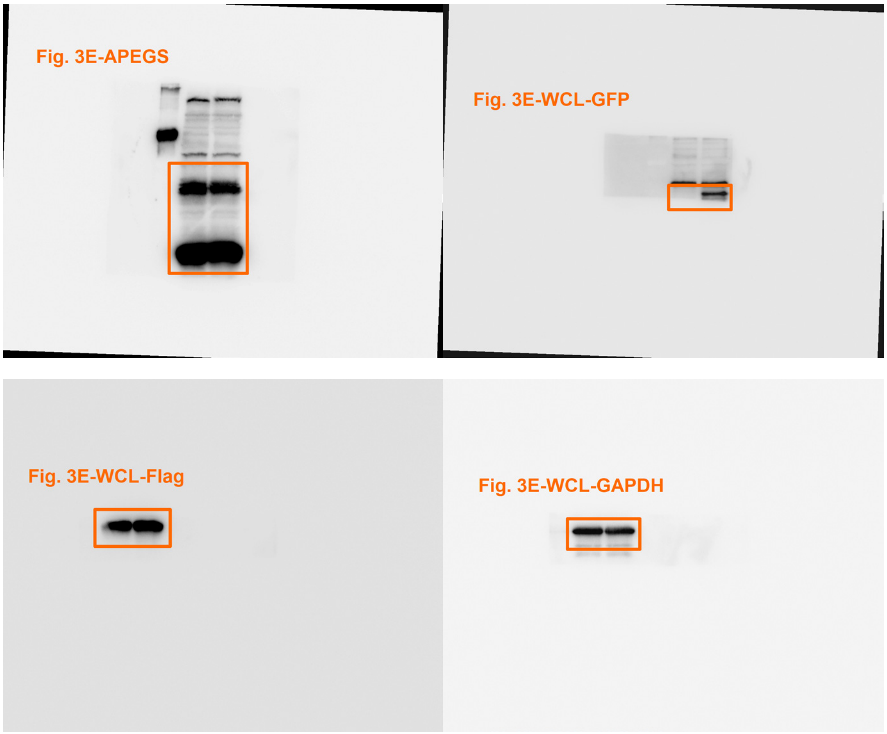
Fig. 3E-APEGS
Fig. 3E-WCL-GFP
Fig. 3E-WCL-Flag
Fig. 3E-WCL-GAPDH

Fig. 4C

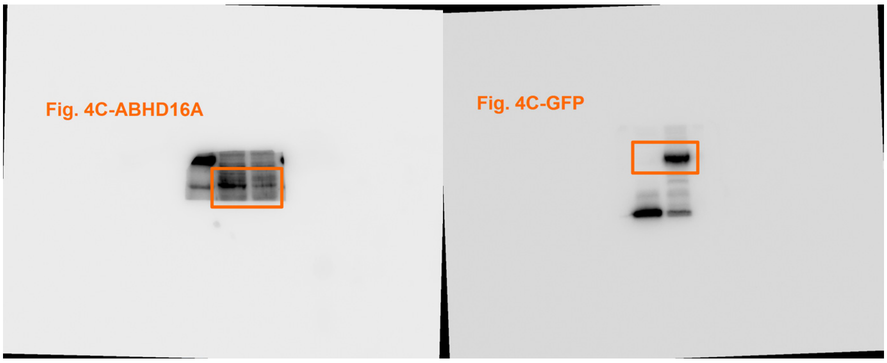
Fig. 4C-ABHD16A
Fig. 4C-GFP

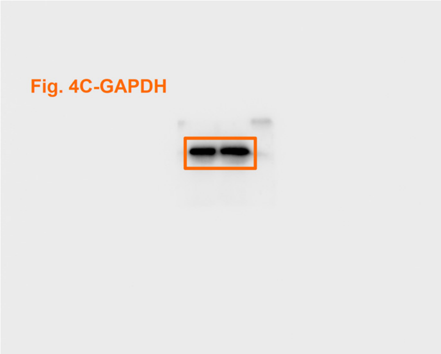
Fig. 4C-GAPDH

Fig. 4E

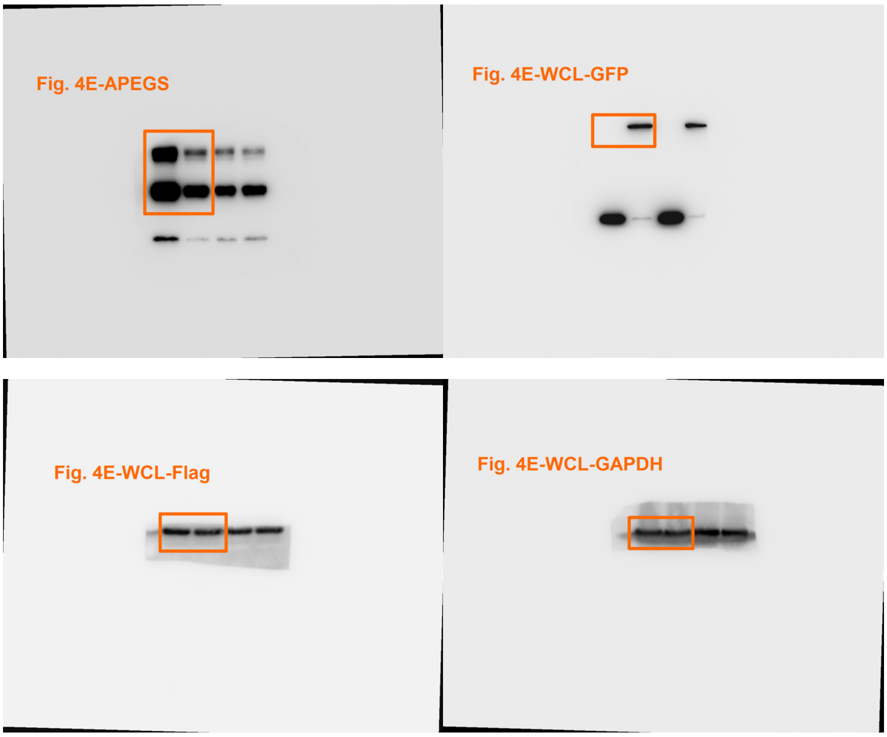
Fig. 4E-APEGS
Fig. 4E-WCL-GFP
Fig. 4E-WCL-Flag
Fig. 4E-WCL-GAPDH

Fig. 4G

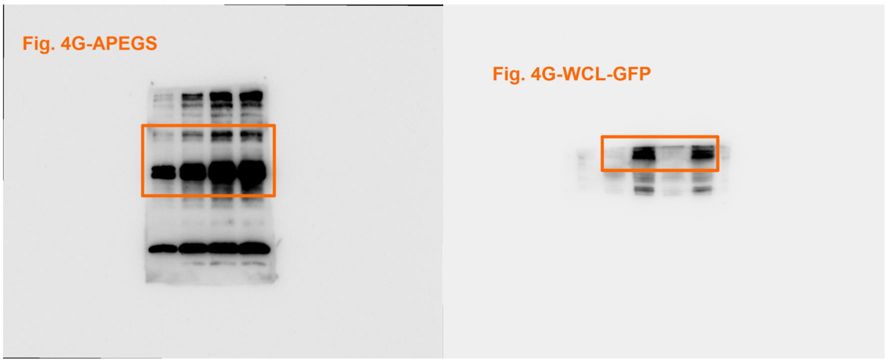
Fig. 4G-APEGS
Fig. 4G-WCL-GFP

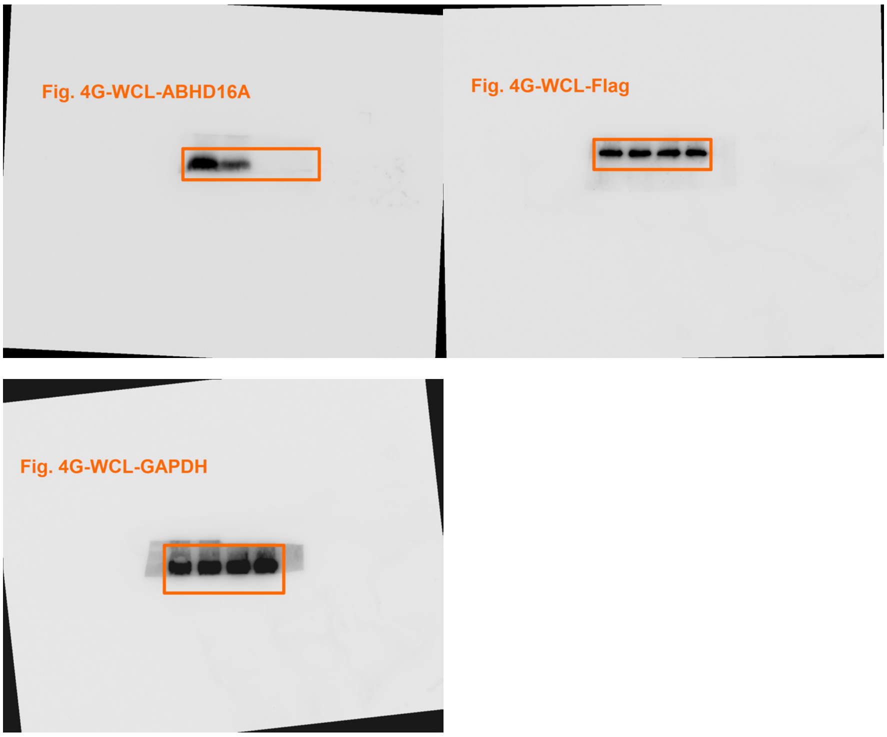
Fig. 4G-WCL-ABHD16A
Fig. 4G-WCL-Flag
Fig. 4G-WCL-GAPDH

Fig. 5B

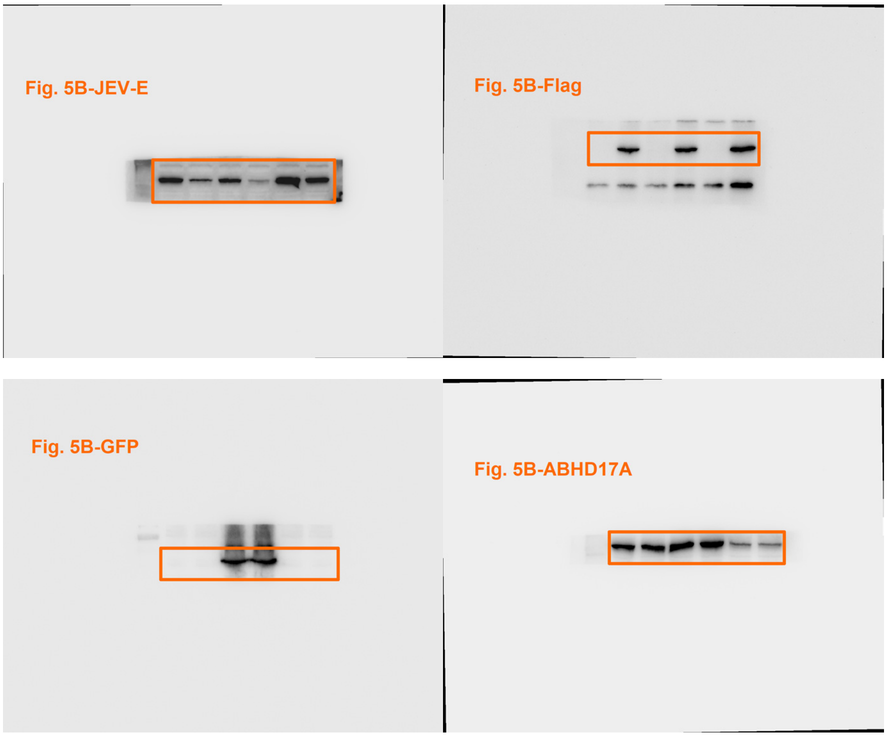
Fig. 5B-JEV-E
Fig. 5B-Flag
Fig. 5B-GFP
Fig. 5B-ABHD17A

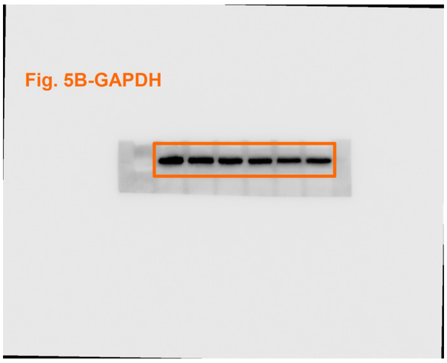

Fig. 5E

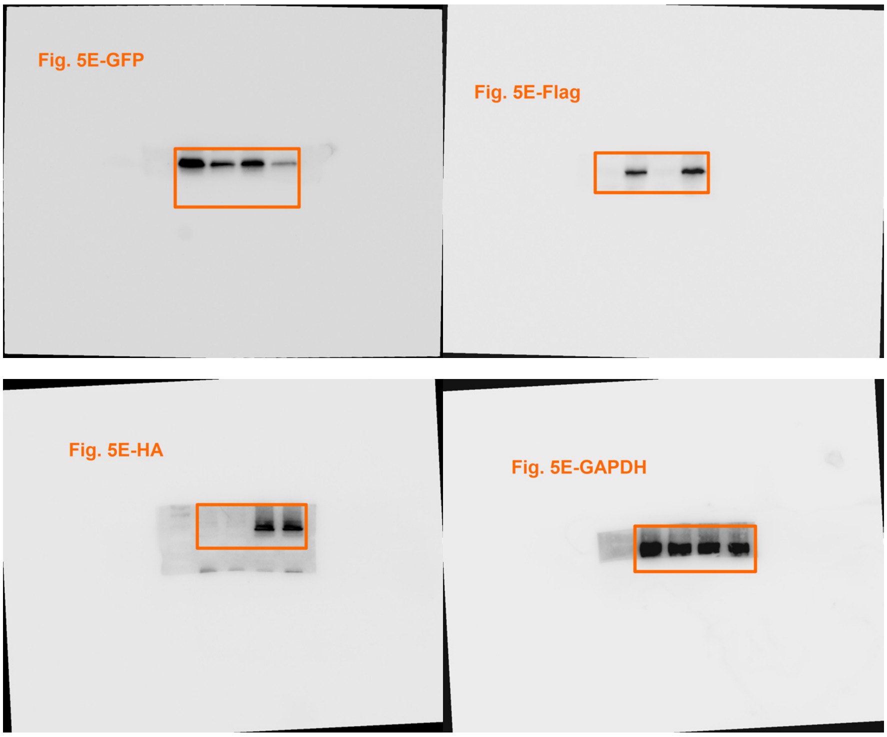

Fig. S1A

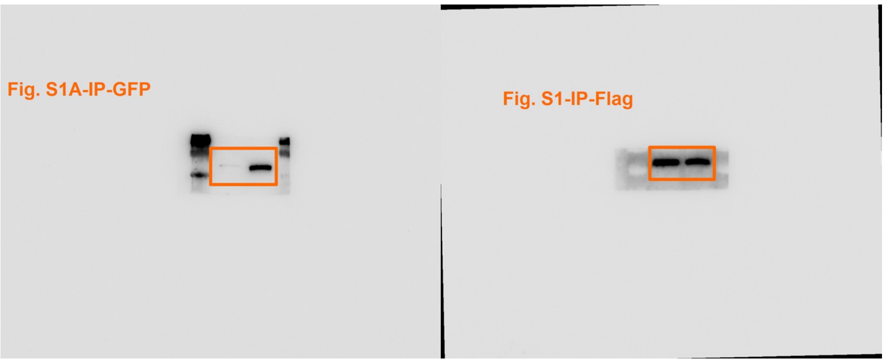

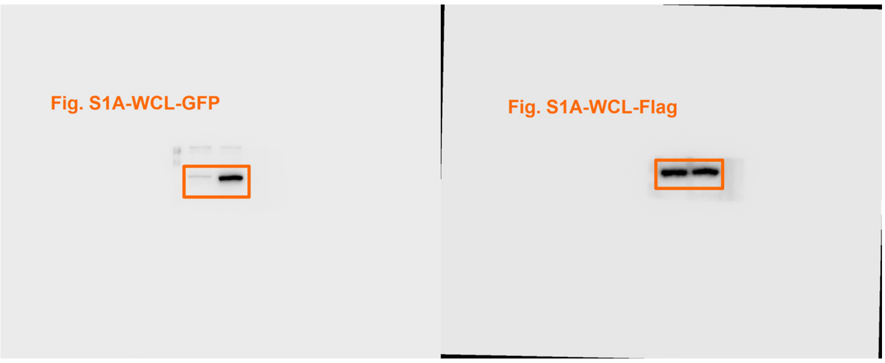

Fig. S1B

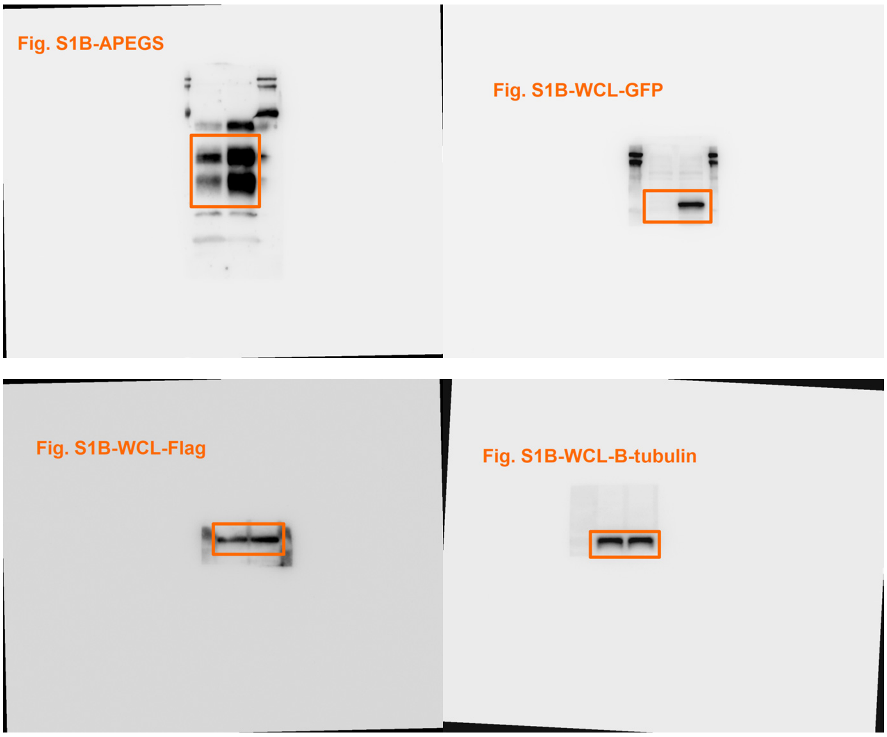

Fig. S2B

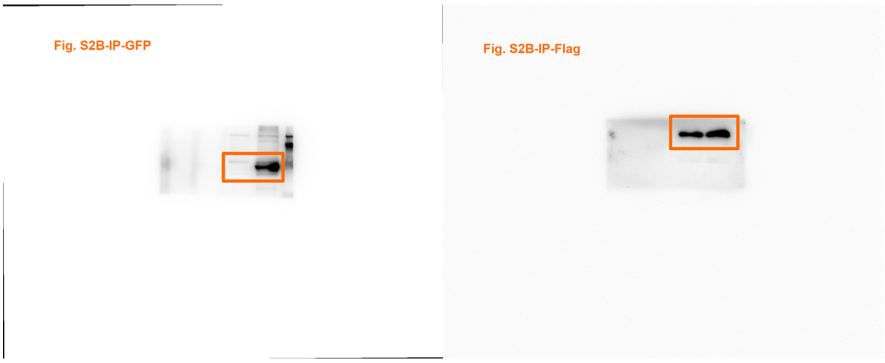

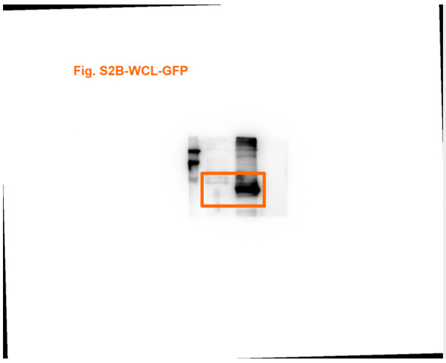
Fig. S2B-WCL-GFP

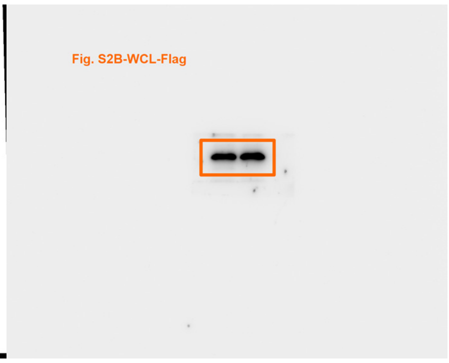
Fig. S2B-WCL-Flag

Fig. S3A

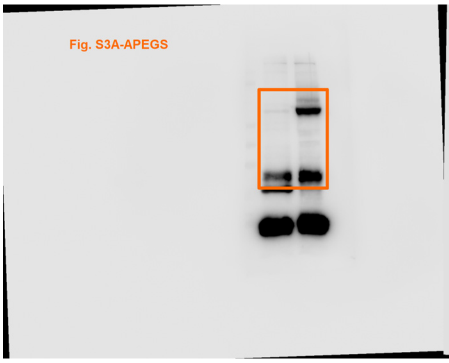
Fig. S3A-APEGS

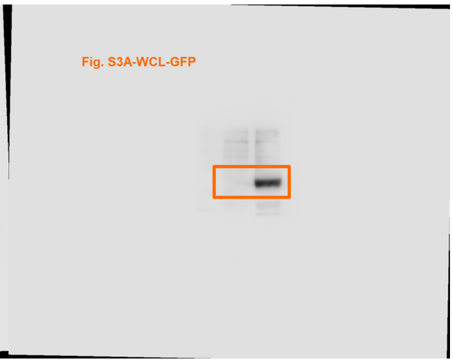
Fig. S3A-WCL-GFP

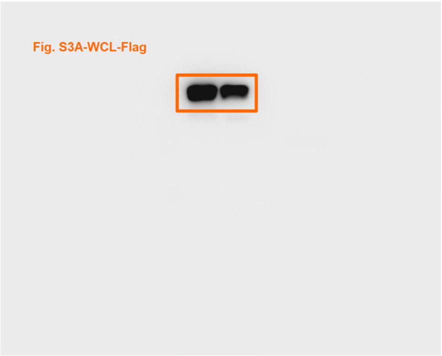
Fig. S3A-WCL-Flag
